# Immigration as the main driver of population dynamics in a cryptic cetacean

**DOI:** 10.1002/ece3.9806

**Published:** 2023-02-11

**Authors:** Simone Tenan, Aurelie Moulins, Paola Tepsich, Alessandro Bocconcelli, Alessandro Verga, Marco Ballardini, Barbara Nani, Daniela Papi, Gabriella Motta, Ana Sanz Aguilar, Massimiliano Rosso

**Affiliations:** ^1^ Institute of Marine Sciences (CNR‐ISMAR) National Research Council Venezia Italy; ^2^ CIMA Research Foundation Savona Italy; ^3^ Advanced Ocean Physics Engineering Woods Hole Oceanographic Institution Woods Hole Massachusetts USA; ^4^ Golfo Paradiso srl Camogli (GE) Italy; ^5^ bluWest Imperia Italy; ^6^ Consorzio Liguria Via Mare Genoa Italy; ^7^ Animal Demography and Ecology Unit, GEDA ‐ IMEDEA (CSIC‐UIB) Esporles Spain; ^8^ Applied Zoology and Conservation Group University of the Balearic Islands Palma Spain

**Keywords:** Cuvier's beaked whale, fecundity, integrated population model, interbirth interval, non‐stationary, *Ziphius cavirostris*

## Abstract

Empirical evidence about the role and interaction of immigration with local demographic processes in shaping population dynamics is still scarce. This knowledge gap limits our capability to derive a conceptual framework that can be used to inform conservation actions. Populations exposed to nonstationary environment do not converge to a stable stage distribution, implying the need for evaluating the demographic role of both vital rates and stage distribution using appropriate tools. This is particularly important for species with larger generation times like cetaceans. We explored the relative demographic role of vital rates and population structure of a poorly known cetacean, the Mediterranean Cuvier's beaked whale, while accounting for the exposure to nonstationary environments. We performed a retrospective analysis through transient life table response experiments (tLTRE) using demographic rates and population structure of both sexes obtained from an integrated population model. The contribution of immigration to variation in realized population growth rates was 4.2, 7.6, and 12.7 times larger than that of female apparent survival, proportional abundance of breeding females with a 2‐year‐old calf, and proportional abundance of breeding females with a 3‐year‐old calf, respectively. Immigration rate and proportional abundance of breeding females with a 2‐ or 3‐year‐old calf explained, respectively, 65% and 20% of total temporal variability in realized population growth rates. Changes in realized population growth rate between successive years were mainly driven by changes in immigration and population structure, specifically the proportional abundance of breeding females with a 2‐year‐old calf. Our study provides insight into the demographic processes that affect population dynamics and in a cryptic cetacean. We presented an analytical approach for maximizing the use of available data through the integration of multiple sources of information for individuals of different distinctiveness levels.

## Graphical abstract

Our study provides insight into the immigration and reproductive processes driving population dynamics of a poorly known cetacean, through an analytical approach that maximizes available data, and can be used to better understand the demographic contribution of life‐history traits and population structure and, ultimately, the persistence of cetacean populations.

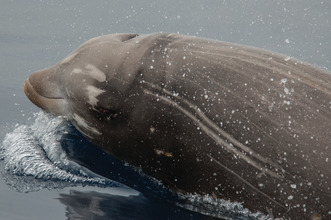

## 1 INTRODUCTION

Understanding the demographic consequences of changes in vital rates and population structure is important to assess factors that can threaten the viability of animal populations and to improve conservation actions (Morris & Doak, 2002). However, information on birth and survival is available only for 1.3% of the tetrapod species, and for 65% of threatened tetrapods we have no information about their vital rates (Conde et al., 2019). The sensitivity of population growth to changes in vital rates is affected by species' life history, with long‐lived species that mature late and produce few or a single offspring, like cetaceans, that are expected to invest more in survival than in reproduction (Brault & Caswell, 1993; Sæther & Bakke, 2000; Young & Keith, 2011). Despite many studies have shown a greater proportional sensitivity of population growth rates to adult survival in long‐lived species, this has been questioned considering that other vital rates, like juvenile/immature survival and reproduction, can show larger temporal variability that may translate into a greater impact on population dynamics (Brault & Caswell, 1993; Manlik et al., 2016; Mills & Lindberg, 2002; Sergio et al., 2011).

Obtaining reliable estimates of demographic rates is critical for evaluating population dynamics and viability but, even when individual longitudinal data to estimate survival are available, the collection of additional data on fecundity rates and immigration can be difficult in hard‐to‐study species. The overall increased availability of data and analytical tools in the past decade has improved our ability to disentangle factors affecting the dynamics of animal populations. Among these factors, immigration is notoriously difficult to record and quantify in wild animal populations (Williams et al., 2002). Quantitative estimates of immigration rates have been made available only recently for some bird and mammal species, for which this vital rate has been confirmed as a key demographic parameter (Millon et al., 2019). While dispersal processes, emigration and immigration, have been recognized as important drivers of metapopulation persistence and local population dynamics (Hanski, 1999), empirical evidence about the role and interaction of immigration with local demographic processes in shaping population dynamics is still scarce, even for the most studied avian and mammalian populations. This empirical knowledge gap limits our capability to derive a conceptual framework that can be used to inform conservation and population ecology (Millon et al., 2019).

Immigration can be estimated through genetic analyses (Rannala & Mountain, 1997; Wang & Whitlock, 2003) or, more precisely, through demographic methods using data on marked animals at multiple populations or through a joint analysis of multiple data types. The latter is performed within an integrated population modeling framework (IPM; Abadi, Gimenez, Arlettaz, et al., 2010; Abadi, Gimenez, Ullrich, et al., 2010; Besbeas et al., 2002) that incorporates a common likelihood typically for population counts, fecundity, and encounter‐reencounter data. In this case, information about immigration and its temporal variability contained in population count data can be derived because the other data sources contain information about the remaining vital rates, and because the IPM framework ensures an adequate representation of errors of the immigration parameter (Millon et al., 2019).

The joint use of estimates of demographic parameters from IPMs and transient life table response experiments (tLTRE; Koons et al., 2016, 2017) has improved in the past few years our understanding about the relative demographic role of vital rates and population structure while accounting for the fact that populations are exposed to changing conditions, that is, nonstationary environments. Empirical evidence derived from the latter framework is, however, limited to a few species and case studies, mainly regarding bird populations (Koons et al., 2017; Nater et al., 2021; Schaub & Ullrich, 2021; Tenan et al., 2021). To our knowledge, no studies on marine mammals employed tLTREs to explore the demographic contribution of vital rates and population structure under nonstationary environmental conditions. A practical limitation of applying tLTREs is in fact the need for estimates of both vital rates and population structure, the latter being difficult to infer for many vertebrates. Here, we used multiple data types integrated into an IPM and tLTREs to study the population dynamics of an endangered cetacean, the Cuvier's beaked whale (*Ziphius cavirostris*). Beaked whales are the least understood among large mammals at a global scale and are particularly vulnerable to anthropogenic noise disturbances caused by geological and seismic surveying, military sonar, and naval traffic that can cause mass strandings (Li & Rosso, 2021). The paucity or lack of baseline data on distribution, population size and structure, and life history of beaked whales limits or impedes quantifying anthropogenic impacts at the population level and design evidence‐based conservation plans (Hooker et al., 2019). The Cuvier's beaked whale is, however, one of the few beaked whale species for which long‐term encounter‐reencounter data from photo‐identification are available and referred to some populations (Curtis et al., 2021; Hooker et al., 2019). The joint analysis of encounter‐reencounter data and population count data allowed us to estimate latent parameters, like immigration, derive multiple fecundity metrics, and explore the relationship between vital rates and population structure and the growth rate of a Mediterranean Cuvier's beaked whale population.

## 2 MATERIALS AND METHODS

### 2.1 Study species and data collection

The Cuvier's beaked whale is a medium‐sized toothed whale with cosmopolitan distribution, and the only beaked whale species commonly found in the Mediterranean Sea. Cuvier's beaked whale has the deepest (2992 m; Schorr et al., 2014) and longest (222 min; Quick et al., 2020) recorded foraging dives among marine mammals. In the Mediterranean, Cuvier's beaked whales occur in deep waters (>200 m) and are often found over the continental slope (Moulins et al., 2007) where they feed primarily on mesopelagic cephalopods (Santos et al., 2001). Genetic analysis has indicated a high degree of differentiation between the Atlantic and the Mediterranean population (Dalebout et al., 2005).

Dedicated boat‐based surveys were conducted in the Ligurian Sea (NW Mediterranean Sea) during 16 years, from 2004 to 2019. The area investigated along the years represents a core area for the species in the Mediterranean sea (Moulins et al., 2008; Tepsich et al., 2014) and it extended beyond the 1000‐m isobath, covering an approximate area of 2000 km^2^. The surveys were conducted in Beaufort sea state ≤4 and swell <1 m. For each sighted group the geographical position was recorded and the group was described in situ on the basis of the minimum, the maximum, and the best estimation of the number of individuals. Photographic captures of whales were collected using auto‐focus digital cameras equipped with a 70–300 mm stabilized zoom lens. The sampling protocol was to photograph randomly as many animals as possible in the group, from both right and left sides, whether or not photographs had already been taken of a particular individual. Whenever possible, photographic sequences were taken in order to capture the whole exposed flank of the whales, from the snout to the tail stock (Coomber et al., 2016). Encounter‐reencounter information of all photographed individuals collected during the 16‐year period derived from a total of 233 surveys (mean 14.6, median 18, range 2–22 surveys per year) annually performed from April to the end of October, with 85% of records collected from June to September.

### 2.2 Photo identification and data selection

Each photograph was assigned a quality rating (*Q*‐value) from 1 to 6 (similar to Gowans & Whitehead, 2001), based on the focus, angle of the animal relative to the sensor plane, the proportion of the frames filled by the animal, and the exposure (for details see Rosso et al., 2011). The *Q*‐value was independent of the amount of markings on the individual. Only captures events described by *Q* ≥ 4 photographs were analyzed in this study, since *Q* ≥ 4 images were of sufficient quality to be used in photographic encounter‐reencounter studies in this species (Rosso et al., 2011). Photographs of each whale—from each (photographic) capture event—were compared with photos from previous sightings. Recognition of individuals was achieved through the observation of natural markings (Rosso et al., 2011). If a photograph matched an individual that was already known in the catalog, the photo was assigned the whale's identification number. If not matched, the individual was given a new identification number and added to the catalog. All the photos were analyzed by the last author, who had 15 years of experience in photo‐identification of Cuvier's beaked whales. Photographic collections from left and right sides were maintained separately, although 93% of individuals were photographed on both flanks.

Individual distinctiveness was assigned according to the number and size of marks (i.e., shape, notches, and nicks) taking values of 0 (no marks with no possibility to distinguish individuals even within a few days), 1 (ephemeral marks that allow distinction in the same year but not across multiple years), and 2 (permanent marks that allow distinction on a multiyear basis). Encounter‐reencounter data included individuals with permanent marks (distinctiveness level 2 only), whereas population count data, derived from encounter‐reencounter information (see below), included individuals of distinctiveness levels 1 and 2.

The photographed individuals were sexed whenever possible. An individual was categorized as male when (at some point in the individual own capture history) displaying erupted teeth or showing an average scarring density ≥0.05 along the flank (Coomber et al., 2016). A toothless individual was categorized as female if photographed in close association with a calf (at some point in the individual own capture history). Twenty‐eight individuals were sexed using DNA‐based genetic techniques (see Baini et al., 2020).

Photographed whales were separated in seven different age/reproductive categories (Table 1). Young‐of‐the‐year and calves lack identifying marks, therefore, information about the presence of a young/calf was included in the observation of the resighted (i.e., identified in a photo) mother (see below). In the case of observations of the same individual during multiple surveys of the same year, the related year‐specific information was grouped to keep the most relevant information on the breeding status for females and on stage for males, in order to define the events (sensu Pradel, 2005) and construct the individual encounter‐reencounter histories (see below).

**TABLE 1:** Age and reproductive categories defined for photographed Cuvier's beaked whales.

Category	Description
Newborn	Fetal folds, always swimming in infant position
Young‐of‐the‐year	Dark brown, equal/less than two‐thirds the adult length, typically swimming in infant position with the mother
Calf	Dark brown, evidently longer than two‐thirds the adult length, generally swimming in infant position or close association with the mother
Juvenile	Dark brown, shorter than adult length, usually showing a paler coloration on a part of the melon
Subadult or prebreeder	Brown, around adult length with pale coloration of the melon that extends backward covering also the blowhole area (this may not apply to some females)
Adult	Around adult length, pale coloration extending along the whale back
Reproductive male	Adult showing erupted teeth
Reproductive female	Adult in close association with a young‐of‐the‐year or a calf

### 2.3 Demographic data

Two types of demographic data were modeled: encounter‐reencounter data and population count data. The encounter‐reencounter dataset included a total of 124 unique individuals (81 males and 43 females; Table S5 in Appendix S1) with 136 and 329 resightings for females and males, respectively (Tables S6 and S7 in Appendix S1); only one individual with unknown sex was discarded from the analysis. Population count data were composed by annual sex‐ and/or stage‐specific counts of the total number of individuals observed in the population in 2004–2019, considering individuals with both permanent and ephemeral marks (distinctiveness levels 1 and 2), thus exploiting information of individuals without long‐lasting marks that is usually discarded in the analysis of multiyear cetacean data based on individual identification. Specifically, counts of juveniles, nonreproductive individuals (i.e., immatures that have not yet recruited as breeders, excluding juveniles, and nonbreeders that have reproduced at least once in the past), and reproductive individuals (breeding females and adult toothed males) were considered. Given the presence of individuals of unknown sex for juveniles and nonreproductive individuals, count data for these two categories were not sex‐specific and included counts of the ensemble of both sexes and individuals with unknown sex, in order to exploit all available information (Section S.1.1, Tables S3 and S4 in Appendix S1). Information on fecundity was embedded in the encounter‐reencounter data, through information of young‐of‐the‐year/calf‐mother association included in the defined event codes (see below).

### 2.4 Statistical analysis

Encounter‐reencounter data and population count data were jointly analyzed to obtain consensual estimates of demographic rates and population sizes, using an integrated population model (IPM, e.g., Besbeas et al., 2002; Schaub & Abadi, 2011; Schaub & Kéry, 2021). The IPM framework allows the estimation of latent demographic parameters, for which no or very few explicit data are available, such as immigration (Abadi, Gimenez, Ullrich, et al., 2010; Schaub & Fletcher, 2015). Inference was based on a joint likelihood derived by the multiplication of the single‐dataset likelihoods described below.

### 2.5 Modeling of population count data

A sex‐specific stage‐structured stochastic population model, that describes changes in population size over time as a function of demographic rates, was used in a state‐space modeling approach (De Valpine & Hastings, 2002), where the observation process that generated count data is conditional on the state process. The stages for females were juvenile, prebreeding, breeding (with a young‐of‐the‐year, 1‐, 2‐, or 3‐year‐old calf), nonbreeding, and (juvenile) immigrant (see below). The stages for males were juvenile, subadult, not toothed adult, toothed adult, and immigrant. Changes in the sex‐ and stage‐specific population sizes between year *t* and *t* + 1 were modeled with Binomial and Poisson processes (Equations S1–S45 and S48–S53 in Appendix S1). The number of immigrants in year *t* was $N_{\mathrm{Imm},\;\mathrm{FM},t}∼\text{Pois}\left(\omega_{t}\right)$ where *ɷ*_*t*_ is the expected number of immigrants (including both sexes) in year *t* (Schaub & Fletcher, 2015). Immigration (*ɷ*_*t*_) was modeled with random year effects and independently of other vital rates over time (Equation S47 in Appendix S1). The expected number of immigrants was not sex‐specific to reduce bias in the estimation of this latent parameter, assuming even sex ratio among immigrants (Equations S13 and S35 in Appendix S1). In addition, immigration was assumed to occur in the juvenile stage. Sex‐ and stage‐specific probabilities were defined for female apparent survival (*ɸ*_*F,t*_, equal for all stages; Section S.1.1 and S.3 in Appendix S1) from year *t* to *t* + 1, young‐of‐the‐year and calf survival ($\phi_{\mathrm{By},F,t}$, conditional on mother survival $\phi_{F,t}$), and the probability of remaining with the mother for a calf from year *t* to *t* + 1 (*F*_*t*_, conditional on survival $\phi_{\mathrm{By},F,t}$ and fixed to 1 for young‐of‐the‐year and 1‐year old calves; Figure S1 in Appendix S1). Note that in the multievent model for encounter‐reencounter data (see below) we modeled the apparent survival probability, $\phi_{\mathrm{Bc},\;F,t}$, for 1‐ and 2‐year‐old calves that remain with the mother up to the third year of life. However, apparent survival probability for 2‐year‐old calves may be biased since true mortality cannot be distinguished from calf emancipation from its mother (Couet et al., 2019). In the state‐space model we thus assumed that true survival for a 2‐year old calf was equal to $\phi_{\mathrm{By},t}$, i.e., survival of young‐of‐the‐year and 1‐year‐old calves that cannot yet emancipate, which gives (conditional on mother survival) $\phi_{\mathrm{Bc},F,t}=\phi_{\mathrm{By},F,t}\;F_{t}$, and probability of remaining with the mother $F_{t}=\frac{\phi_{\mathrm{Bc},F,t}}{\phi_{\mathrm{By},F,t}}$ (see Appendix S1 for further details). Time‐invariant transition probabilities from juvenile to prebreeding female ($\psi_{\text{JuvPB},F}$) and from prebreeding to breeding female ($\psi_{\text{PbBy},F}$, i.e., probability of first reproduction) were also defined, along with the time‐variant breeding probability for nonbreeding females (*γ*_*t*_). The model also included the apparent survival probability for juvenile and subadult males from year *t* to *t* + 1 ($\phi_{\text{JuvSub},M,t}$), the apparent survival probability for adult males (both not toothed and toothed; $\phi_{\mathrm{Ad},M,t}$) and the stage transition probability ($\psi_{M}$, equal among stages and time invariant, following the best model structure selected for the encounter‐reencounter data, see below).

The observation model described the relationship between annual counts of juveniles, nonreproductive individuals (i.e., immatures that have not yet recruited as breeders, excluding juveniles, and nonbreeders that have reproduced at least once in the past), breeding females, and adult toothed males with true sex‐ and stage‐specific population sizes using Binomial processes with detection probabilities obtained from the multievent model (Equations S54–S57 in Appendix S1). Detection probabilities were shared between the two submodels given that the observation process for count data was the same as for photo‐identification encounter‐reencounter data. This, however, implies the assumption that sex‐ and stage‐specific detection probability does not change between individuals with distinctiveness levels 1 and 2.

### 2.6 Modeling of encounter‐reencounter data

Individual encounter histories were modeled using sex‐specific multievent capture‐recapture models (Pradel, 2005), in order to accommodate uncertainty in state assignment. We assumed eight states and seven types of mutually exclusive events that could be observed for females, and five states and six event types for males (Table 2). We also assumed that all female breeding states were ascertained with certainty.

**TABLE 2:** States and codes of mutually exclusive events that could be observed implemented in the multievent model for encounter‐reencounter data of the Cuvier's beaked whale population.

Female		Male	
States			
*Juv*	Juvenile	*Juv*	Juvenile
*Pb*	Prebreeder	*Sub*	Subadult
*By*	Breeder with a young‐of‐the‐year	*AdNt*	Not toothed adult male
*Bc*1	Breeder with a 1‐year‐old calf	*Ad*	Toothed adult male
*Bc*2	Breeder with a 2‐year‐old calf	*D*	Dead
*Bc*3	Breeder with a 3‐year‐old calf		
*Nb*	Nonbreeder		
*D*	Dead		
Events			
1	Not seen	1	Not seen
2	Seen as juvenile	2	Seen as juvenile
3	Seen as prebreeder	3	Seen as subadult
4	Seen as breeder with a young‐of‐the‐year	4	Seen as not toothed adult
5	Seen as breeder with a 1‐year‐old or older calf	5	Seen as toothed adult
6	Seen as nonbreeder	6	Seen as adult^a^
7	Seen alone^b^		

^a^ Event ‘6’ for males could include either a not toothed or a toothed adult, being this feature not always ascertained.

^b^ Event ‘7’ for females could include either a prebreeder or a nonbreeder that may be difficult to distinguish based on coloring.

Probabilities of survival for females ($\phi_{F}$), young‐of‐the‐year ($\phi_{\mathrm{By},F}$), and calves ($\phi_{\mathrm{Bc},F}$), as well as breeding probability (*γ*) where modeled with random year effects and a variance–covariance matrix for temporal correlation between the parameters (Equations S62–S66 in Appendix S1). Similarly to female probabilities, male apparent survival probabilities ($\phi_{\text{JuvSub},M}$ and $\phi_{\mathrm{Ad},M}$) were also modeled with random year effects and with a temporal correlation structure (Equations S67–S69 in Appendix S1). Temporal random effects were used for modeling encounter probabilities of females and males, in addition to the effect of year‐specific sampling effort in both sexes and a stage‐specific effect on male probabilities (Equations S70 and S71 in Appendix S1). For further details about the multievent model structure and selection see Appendix S1.

### 2.7 Fecundity

Fecundity metrics embedded in the IPM are the time‐variant breeding probability for a nonbreeding female (*γ*_*t*_), the interbirth interval derived as $\left(1/\hat{\gamma }\right)+1$, where $\hat{\gamma }$ is the average breeding probability, the probability of first reproduction ($\psi_{\text{PbBy},\;F}$, i.e., the time‐invariant probability of moving from the prebreeding to the breeding female state), and the weaning probability (i.e., the complement, 1−*F*_*t*_, of the probability of remaining with the mother for a calf of 2–3 years of age, from year *t* to *t* + 1, conditional on calf survival $\phi_{\mathrm{By},\;F,t}$). In addition to these quantities, another two were derived taking advantage of the integrated population modeling framework. The reproductive rate (RR_*t*_), expressed as the proportion of females within the study area that have a young‐of‐the‐year among those that have already recruited as breeder (i.e., have already reproduced at least once):
(1)

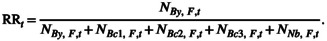




In addition, the weaning rate per female (WR_*t*_) was derived from the breeding female apparent survival probability (*ɸ*_*F*_), the young‐of‐the‐year/calf survival ($\phi_{\mathrm{By},\;F}$; conditional on breeding female apparent survival), and the probability for a calf of remaining with the mother (*F*) until the third year of life, considering the two possible scenarios for a calf to be weaned between 2 and 3 years of age (first row of Equation 2) or at 3 years of age (second row of Equation 2):
(2)

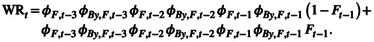




### 2.8 Bayesian inference

The integrated population model was fit using a Bayesian formulation with Markov chain Monte Carlo simulations. Vague prior distributions were used for all parameters (see supplementary model code for details on prior specification in Appendix S1). Summaries of the posterior distribution were calculated from 80,000 posterior samples (burn‐in = 5,000,000 iterations). We assessed convergence using the $\hat{R}$ diagnostics (Brooks & Gelman, 1998) that was <1.02 for all parameters. Posterior predictive checks were performed to assess the goodness‐of‐fit of the different (sub)models (see Appendix S1). Models were implemented using the R (R Core Team, 2012) package jagsUI (Kellner, 2016).

### 2.9 Demographic influence on population growth rate

We performed a retrospective analysis to understand population dynamics in relation to changes in demographic rates of both sexes (female and young‐of‐the‐year/calf survival, breeding probability, probability for a calf of remaining with the mother until 3 years of age, survival probability for juvenile and subadult males, survival probability for adult males, both not toothed and toothed, and immigration rate) and the proportional stage‐structured and sex‐specific population sizes (${\tilde{N}}_{\text{stage},\mathrm{sex}}$, evaluated at temporal mean values) through transient life table response experiments (tLTRE; Koons et al., 2016, 2017) that account for the fact that populations are exposed to nonstationary environments. Population growth rate can be expressed as $\lambda_{t}=N_{t+1}/N_{t}=\|N_{t+1}\|/\|N_{t}\|=\|A_{t}N_{t}\|/\|N_{t}\|$, where ‖‖ denotes the sum of absolute values of vector elements (i.e., the 1‐norm), ***A***_***t***_ is the Leslie matrix and ***N***_***t***_ is the population vector in year *t*. For details about the product of the Leslie matrix and the population vector see Appendix S1 (Section S.2).

The first tLTRE measures the contribution of temporal variability in demographic rates and population structure to the temporal variance of realized population growth rate *λ*_*t*_. If we define vector *q*_*t*_ whose elements are the demographic rates and the proportional stage‐structured population sizes ($\tilde{N}$), where proportional means that they sum to one for each year, the contribution of variability in each demographic rate *θ*_*i*,*t*_ and each component of structured abundance *θ*_*j*,*t*_ to the temporal variance of *λ*_*t*_ is:

(3) $${\left.{\text{Contribution}}_{\theta_{i}}^{\mathrm{var}\left(\lambda_{t}\right)}=\sum_{j}\mathrm{cov}\left(\theta_{i,t},\theta_{j,t}\right)\frac{\partial \lambda_{t}}{\partial \theta_{i,t}}\frac{\partial \lambda_{t}}{\partial \theta_{j,t}}\right|}_{\bar{\theta }},$$

where sensitivity of *λ*_*t*_ to change in each underlying demographic parameter ($\partial \lambda_{t}/\partial \theta_{i,t}$) or abundance component ($\partial \lambda_{t}/\partial \theta_{j,t}$) is evaluated at the mean of *θ*_*t*_ across the study period ($\bar{\theta }$).

The second tLTRE quantifies the contribution of differences in demographic rates and proportional population sizes to the change in *λ*_*t*_ between successive years (Δ*λ*_*t*_):

(4) $${\left.{\text{Contribution}}_{\theta_{i}}^{\Delta \lambda_{t}}\approx \left(\theta_{i,t+1}-\theta_{i,t}\right)\frac{\partial \lambda_{t}}{\partial \theta_{i,t}}\right|}_{{\bar{\theta }}_{i}}.$$

## 3 RESULTS

**FIGURE 1:**
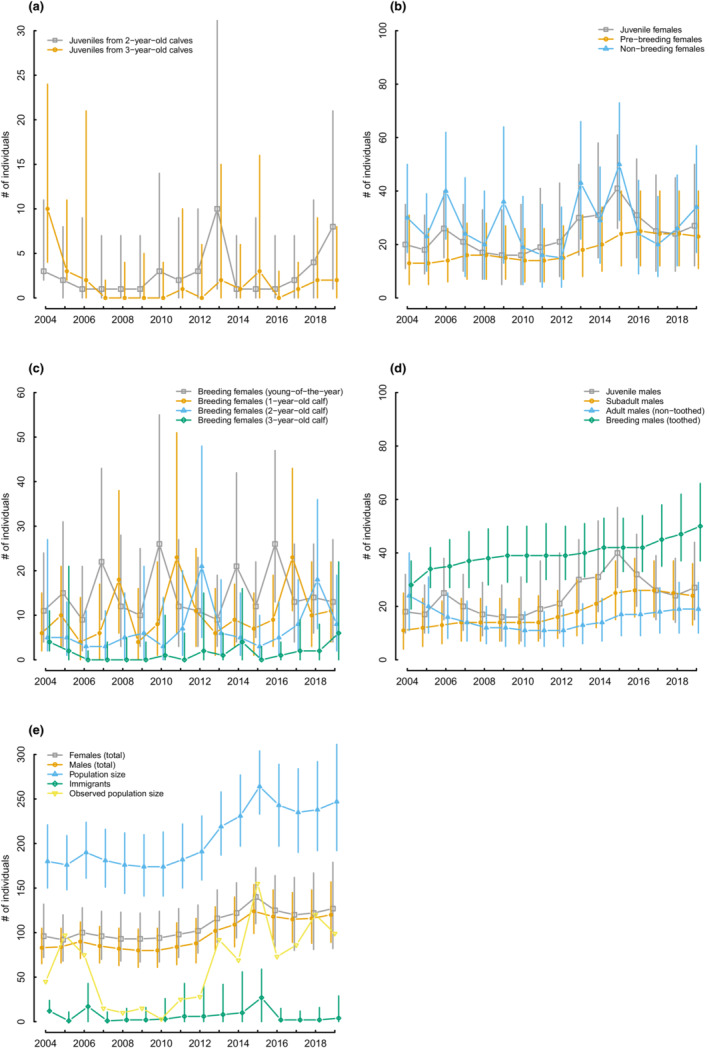
Estimated and observed year‐specific numbers of individuals of the Cuvier's beaked whale population. The vertical lines indicate the 95% credible intervals of the annual estimates.

**FIGURE 2:**
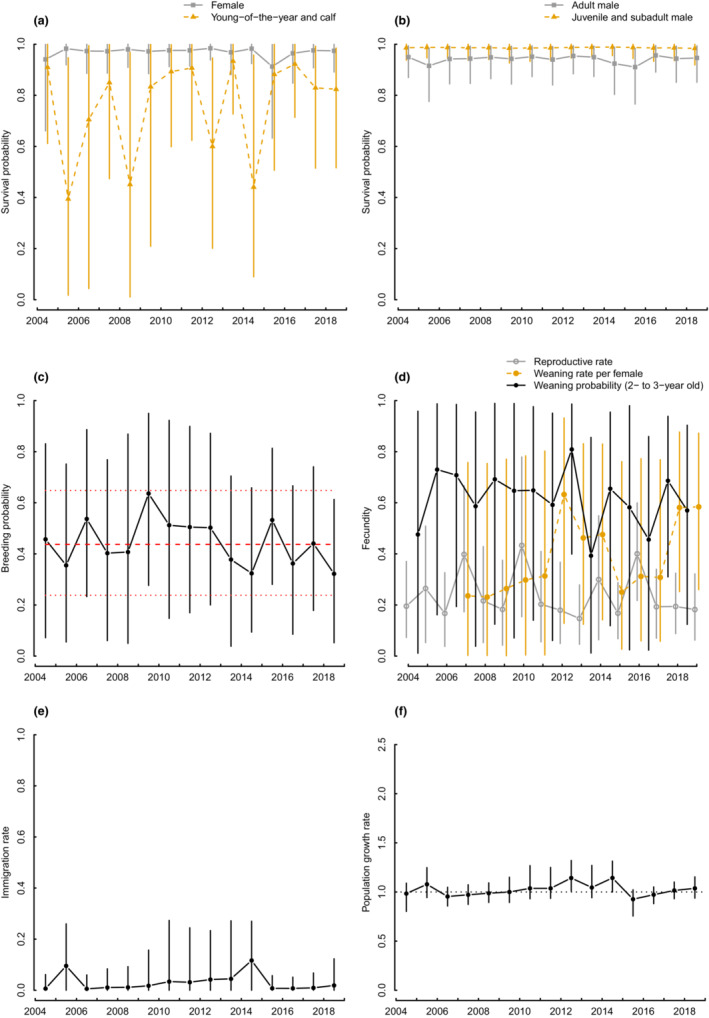
Estimates of year‐specific demographic rates (a–e) and population growth *λ*_*t*_ (f) of the Cuvier's beaked whale population. In (c), the red dashed line indicates the mean from the random effects model, and the red dotted lines indicate the 95% credible intervals of the mean. The vertical lines indicate the 95% credible intervals for the annual estimates. The horizontal black dotted line in (f) indicates population stability.

The estimated Cuvier's beaked whale annual population ranged from 174 (95% CRI 141–210 in 2009, 141–213 in 2010) to 264 individuals (95% CRI 233–304 in 2015) in the period 2004–2019, with an average annual increase of 2.1% but with uncertainty that encompasses population stability (geometric mean of population growth rate *λ* 1.021, 0.998–1.043) (Figures 1 and 2f). Mean apparent survival probability of weaned individuals was the highest for juvenile and subadult males (0.989, 0.963–1.000), intermediate for females (0.980, 0.946–0.998), and the lowest for adult males (both not toothed and toothed; 0.949, 0.915–0.978). Temporal variability of apparent survival of weaned individuals was, on average, larger for females than males, despite 95%CRIs largely overlapped (Figure 2a,b; Table S1 in Appendix S2). Conditional on mother survival, mean young‐of‐the‐year, and calf survival was 0.835 (0.602–0.973) with a temporal variability considerably larger than that of survival of weaned individuals (Figure 2a; Table S1 in Appendix S2). Temporal correlation between demographic rates was generally weak, and 95% CRIs for temporal correlation coefficients always encompassed zero, except for female apparent survival probability and young‐of‐the‐year survival (Tables S2 and S3 in Appendix S2). Breeding probability for nonbreeding females that have reproduced at least once in the past, was on average 0.437 (0.238–0.648) throughout the study period, ranging between 0.322 (0.052–0.613) in 2019 (with a similar minimum value of 0.324 in 2014) and 0.636 (0.277–0.950) in 2009 (Figure 2c). The average breeding probability translates into an interbirth interval of 3.29 years (2.54–5.20). Probability of first reproduction, that is, the time‐invariant transition probability from the prebreeding to the breeding female stage, was 0.245 (0.121–0.425). Weaning probability, for a calf of 2 to 3 years of age, was on average 0.615 (0.050–0.974) across the whole study period, and ranged between 0.393 and 0.809 (Figure 2d). Reproductive rate, that is the proportion of females within the study area that have a young‐of‐the‐year among those that have already recruited as breeder, was on average 0.239 (0.060–0.567) across the study period, and ranged between 0.147 and 0.433 (Figure 2d). Mean weaning rate per female on the entire study period was 0.381 (0.012–0.847) and ranged between 0.231 and 0.633 (Figure 2d). Annual estimates of the immigration rate (both sexes) ranged between 0.006 (0–0.060, with minimum values estimated in 2004, 2006, and 2016) and 0.117 (0–0.270, in 2014), with an average for the whole study period of 0.043 (0–0.216) (Figure 2e).

### 3.1 Demographic influence on temporal variance of population growth

Decomposing the variance of realized population growth rates using transient life table response experiments (tLTRE; Appendix S1, Equation 3) showed that immigration rate contributed most to temporal variability in realized population growth rates (65% of total variation), followed by female apparent survival (13%), proportional abundance of breeding females with a 2‐year‐old calf (12%), and proportional abundance of breeding females with a 3‐year‐old calf (8%). All other parameters contributed less than 3% of total variation each (Table 3).

**TABLE 3:** Estimated sensitivities and elasticities of realized population growth rate to changes in the underlying sex‐specific vital rates, overall immigration rate, and proportional population structure (i.e., stage‐specific proportions of abundance, evaluated at temporal mean values), and transient life stable response experiment (tLTRE) contributions to temporal variability in realized population growth rate, for the Cuvier's beaked whale population in the period 2004–2019.

	Sensitivity			Elasticity			tLTRE contribution		
	Mean	2.5%	97.5%	Mean	2.5%	97.5%	Mean	2.5%	97.5%
Female vital rates									
Apparent survival, *ɸ*_*F*_	0.552	0.472	0.629	0.527	0.446	0.606	0.0009	−0.0002	0.0045
Young‐of‐the‐year/calf survival, $\phi_{\mathrm{By},F}$	0.023	0.012	0.037	0.019	0.008	0.033	−0.0001	−0.0005	0.0001
Breeding probability, *γ*	0.000	0.000	0.000	0.000	0.000	0.000	0.0000	0.0000	0.0000
Prob. remain with the mother, *F*	−0.032	−0.049	−0.017	−0.011	−0.011	−0.011	0.0001	−0.0002	0.0007
Female proportional abundance									
Juvenile, ${\tilde{N}}_{\mathrm{Juv},F}$	−0.022	−0.044	−0.003	−0.002	−0.002	−0.002	0.0000	−0.0000	0.0000
Prebreeding, ${\tilde{N}}_{\mathrm{Pb},F}$	−0.022	−0.044	−0.003	−0.003	−0.003	−0.003	0.0000	−0.0000	0.0000
Breeding (young‐of‐the‐year), ${\tilde{N}}_{\mathrm{By},F}$	−0.022	−0.044	−0.003	−0.002	−0.002	−0.002	−0.0000	−0.0000	0.0000
Breeding (1‐year‐old calf), ${\tilde{N}}_{\mathrm{Bc}1,F}$	−0.022	−0.044	−0.003	−0.002	−0.002	−0.002	−0.0000	−0.0000	0.0000
Breeding (2‐year‐old calf), ${\tilde{N}}_{\mathrm{Bc}2,F}$	0.482	0.268	0.685	0.021	0.021	0.021	0.0005	−0.0001	0.0018
Breeding (3‐year‐old calf), ${\tilde{N}}_{\mathrm{Bc}3,F}$	0.961	0.926	0.986	0.015	0.015	0.015	0.0003	−0.0001	0.0014
Nonbreeding, ${\tilde{N}}_{\mathrm{Nb},F}$	−0.022	−0.044	−0.003	−0.004	−0.004	−0.004	0.0000	−0.0000	0.0001
Male vital rates									
juv/sub apparent survival, $\phi_{\text{JuvSub},M}$	0.208	0.157	0.265	0.220	0.220	0.220	0.0000	−0.0001	0.0002
Adult apparent survival, $\phi_{\mathrm{Ad},M}$	0.272	0.228	0.319	0.265	0.265	0.265	0.0001	−0.0003	0.0007
Male proportional abundance									
Juvenile, ${\tilde{N}}_{\mathrm{Juv},M}$	−0.016	−0.048	0.013	−0.002	−0.002	−0.002	0.0000	−0.0000	0.0000
Subadult, ${\tilde{N}}_{\mathrm{Sub},M}$	−0.016	−0.048	0.013	−0.002	−0.002	−0.002	0.0000	−0.0000	0.0000
Not toothed adult, ${\tilde{N}}_{\text{AdNt},M}$	−0.061	−0.091	−0.031	−0.006	−0.006	−0.006	0.0000	−0.0001	0.0001
Toothed adult, ${\tilde{N}}_{\mathrm{Ad},M}$	−0.061	−0.091	−0.031	−0.014	−0.014	−0.014	−0.0000	−0.0001	0.0000
Immigration rate, $\iota_{\mathrm{FM}}$	1.000	1.000	1.000	0.018	0.018	0.018	0.0038	0.0003	0.0106

### 3.2 Demographic influence on changes in population growth between successive years

**FIGURE 3:**
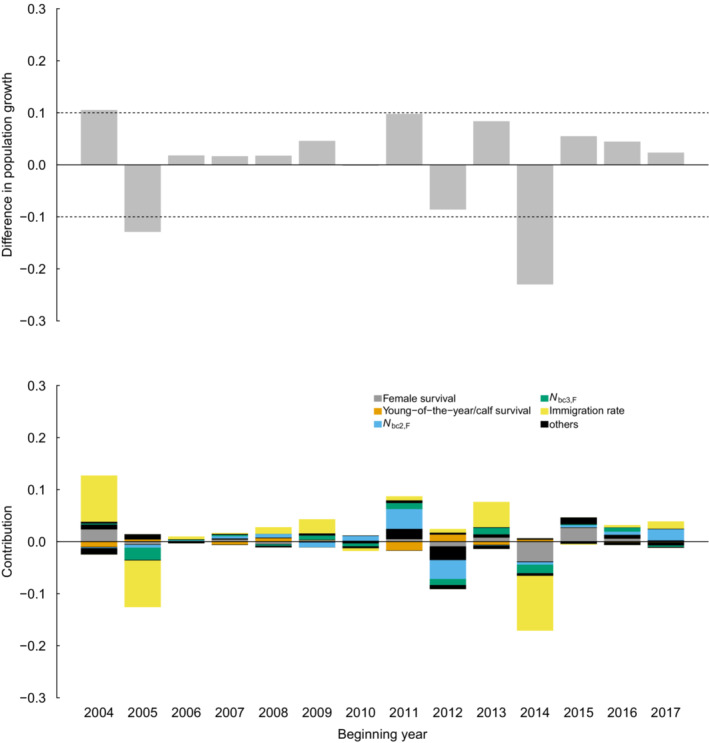
Sequential changes in realized population growth rate for the Cuvier's beaked whale population, and contributions of the changes in demographic rates and population structure. The annual difference in realized population growth rate equals the sum across all contributions. Colors highlight the parameters whose changes contributed the most to at least one change in population growth during the study period; all the other parameters with minor contributions are grouped and reported in black.

The application of tLTRE to changes in realized population growth rate between successive years (Appendix S1, Equation 4), with sequential changes that ranged from −0.23 to 0.11, showing that when population growth rates changed substantially (Δ > 0.1 or Δ < −0.1) the dominant driver was immigration rate in all cases. However, changes in population growth rate greater than ∣0.1∣ occurred only three times (2004–2005, 2005–2006, 2014–2015) during the study period. Considering a slightly lower absolute threshold value of changes in population growth rate (i.e. Δ > 0.08 or Δ < −0.08), which occurred six times during the study period, confirmed immigration rate as the dominant driver (67% of the cases) followed by the proportional abundance of breeding females with a 2‐year‐old calf (33%). All other demographic parameters and components of population structure contributed the least (Figure 3).

## 4 DISCUSSION

We provided for the first time for a beaked whale species estimates of different fecundity (or reproductive) metrics and of immigration, along with other sex‐ and/or stage‐specific vital rates. Survival estimates available for other populations of the Cuvier's beaked whale are not sex and age/stage specific, but on average were similar or lower to our average estimates (0.95 for a Californian population, Curtis et al., 2021, and 0.91 for a Northeast Atlantic population, Reyes Suárez, 2018). We also provided estimates of the breeding probability for a nonbreeding female, the interbirth interval, the probability of first reproduction, the weaning probability (i.e., the complement of the probability of remaining with the mother for a calf of 2–3 years of age, conditional on calf survival), the reproductive rate (expressed as the proportion of females within the study area that have a young‐of‐the‐year among those that have already reproduced at least once), and the weaning rate per female. The large credible intervals for young‐of‐the‐year and calf survival, as well as for reproductive parameter estimates (Figure 2), are a consequence of limited information in the encounter‐reencounter data, and require cautious interpretation of their demographic influence on population growth rate. On the contrary, uncertainty in the estimation of immigration rates is limited and should not affect the assessment of its demographic contribution. In the case of large uncertainty around immigration estimates, a simulation study should be conducted to evaluate how temporal variation and sample size can affect the estimation of the contribution of immigration (Paquet et al., 2021).

Estimates of reproductive parameters are key to assess the status of populations and effectively direct conservation efforts, but in cetaceans these are available only for a small number of populations and have been commonly derived from observed data without accounting for imperfect detection and separating sampling and process variance (Arso Civil et al., 2017; Cheney et al., 2019). Not accounting for imperfect detection of breeding females with young‐of‐the‐year/calf may lead to underestimating fecundity (i.e., reproductive rate) as well as overestimating interbirth interval and survival of young (Cheney et al., 2019). Difficulties in the estimation of fecundity rates and interbirth intervals in cetaceans also arise from shortness of study periods relative to life span, missing data years, low reencounter probabilities, and in some cases the uncertainty in ascertaining the breeding status (e.g., when young is not sighted with the female or die before female encounter; Arso Civil et al., 2017; Cheney et al., 2019). Reproductive status uncertainty in cetaceans has been accounted for only in a few studies (Cheney et al., 2019; Couet et al., 2019; Rankin et al., 2013). In addition, fecundity in cetaceans has been expressed in a number of different ways, like the proportion of young‐of‐the‐year (e.g., Tezanos‐Pinto et al., 2015) or calves (e.g., Craig & Herman, 2000) on the total number of identified individuals in the population of both sexes and including newborn and calves (the so‐called crude birth rates), the proportion of calves among all otherwise unfiltered recapture‐qualifying records (Curtis et al., 2021), the proportion of identifiable reproductive females with newborn or calves on the total number of sexually mature females (called calving rates; e.g., Kogi et al., 2004), the inverse of the interbirth interval (Matkin et al., 2014), by modeling interbirth intervals based on the probability of birth conditional on previous reproductive histories (e.g., Arso Civil et al., 2017), or simply reporting the mean observed interbirth interval (e.g., Henderson et al., 2014). Estimates of interbirth intervals tend to be biased if young‐of‐the‐year/calf encounter probability is not accounted for (Arso Civil et al., 2017), or if the length of the study does not allow the longest birth intervals to be observed (Barlow & Clapham, 1997). Hoyle and Maunder (2004) derived a fecundity rate per mature individual at equilibrium calculated as the inverse of the number of breeders per recruit at carrying capacity, but did not account for imperfect detection. The approach used by Arso Civil et al. (2017) to estimate fecundity rate, based on the probability of a female giving birth conditional on a previous birth, was able to take account for imperfect detection, individual and temporal heterogeneity in re‐sightings. However, neither this nor the most commonly used methods could be used to explore temporal variation in reproductive rates (Cheney et al., 2019). Matkin et al. (2014) estimated the mean age at first reproduction using the observed proportion of females and males maturing at each age, not accounting for imperfect detection and uncertainty in age/stage classes. Rankin et al. (2013) estimated calving intervals and breeding rate considering imperfect detection, but they did not account for uncertainty regarding breeding state assignment. Couet et al. (2019) estimated breeding probability dependent on previous reproductive state while accounting for imperfect detection and uncertainty regarding breeding state assignment using multievent models. Another recent paper by Cheney et al. (2019) employed multistate models to estimate the proportion of females within the study area that have a newborn calf (referred as the unconditional reproductive rate) and the transition probability from nonbreeder to female with a newborn (referred as conditional reproductive rate). In our study the unconditional reproductive rate is similar to the one reported by Cheney et al. (2019) but based on the part of the population of individuals that have reproduced at least once (in accordance with Jacobson et al., 2020) and not based on the total number of females in the population, that includes also immature females. Breeding probability for nonbreeding females (*γ*_*t*_) in our study corresponds to the conditional reproductive rate of Cheney et al. (2019). Similarly to Cheney et al. (2019), we provide time‐varying estimates of fecundity while accounting for imperfect detection, but the integrated modeling framework allowed us to derive additional fecundity metrics. However, we note that our fecundity estimates cannot account for neonate mortality or mortality of young‐of‐the‐year/calf before first encounter, since females were not classified as breeders, thus having given birth, if their young‐of‐the‐year/calf was never sighted with them.

Only a few studies integrated different types of data to improve estimates of demographic parameters of cetaceans. Mosnier et al. (2015) integrated count data and hunting catches (but no encounter‐reencounter data) and estimated a pregnancy rate among mature females available for reproduction (i.e., females not pregnant, not with a calf <1 year‐old, or that have lost a calf during the previous year). Jacobson et al. (2020) used an integrated population model (IPM) with density‐dependent fecundity, that is, with a parameter that controls how fecundity changes as the population approaches carrying capacity. Boyd and Punt (2021) integrated distance‐sampling and encounter‐reencounter data and derived an average per‐capita fecundity from the proportion of cow‐calf pairs among detected animals. The flexibility of the IPM framework here used applies also to the immigration parameter itself, which can be expressed and modeled as a rate relative to previous population size or directly as the number of immigrating individuals (Schaub & Fletcher, 2015) like in our case. Immigration is a latent parameter, and we assumed it occurred at the juvenile stage and with even sex ratio among immigrants in order to reduce estimation bias and not further complicate the model due to the limited data available to inform the life‐history parameter estimates. To our knowledge, estimates of immigration in cetaceans are rare and we are aware of only one study reporting the proportion of immigrants for an Humpback whale population (0.014–0.027) (Cypriano‐Souza et al., 2017) and no previous estimates for the Cuvier's beaked whale or other beaked whale species.

### 4.1 Demographic influence on population growth

We performed a retrospective analysis through transient life table response experiments (tLTRE; Koons et al., 2016) using demographic rates and population structure of both sexes derived from the IPM to assess their relative demographic role. To our knowledge, no previous studies on cetaceans employed tLTREs. More generally, tLTRE are important because they allow identifying which parameters have driven temporal variation in population growth rate and have thus determined the dynamics of the population, in the presence of nonstationary environments. We focused on realized population growth rates, as opposed to asymptotic growth rates that require assumptions like stationary environment and a stable stage distribution, which are rare in natural conditions and populations experiencing anthropogenic pressures (Schaub & Kéry, 2021). Populations that are exposed to a nonstationary environment remain in a permanent transient state with no chance to converge to a stable stage distribution (Hastings, 2004). Therefore, populations that are not in a stable stage distribution depends not only on demographic rates but also on the actual stage distribution (Schaub & Kéry, 2021). In addition, in species with longer phases of transient dynamics, for example, species with larger generation times like cetaceans, the impact of population structure becomes stronger and the results of transient and classical LTRE will differ more (Koons et al., 2016). Transient LTREs should thus be preferred in demographic studies on cetaceans.

In our study population, the contribution of immigration to variation in realized population growth rates was 4.2, 7.6, and 12.7 times larger than female apparent survival, proportional abundance of breeding females with a 2‐year‐old calf, and proportional abundance of breeding females with a 3‐year‐old calf, respectively. Immigration rate (average 0.043) affected the most temporal variability in realized population growth rates (65% of total variation). Proportional abundance of breeding females with a 2‐ or 3‐year‐old calf explained 20% of temporal variation in realized population growth rates, suggesting that local recruitment is another important driver of population dynamics of this cetacean. Note that weaning probability for 2‐year‐old calves was on average 0.615 across the whole study period, suggesting that the majority of calves weaned before reaching their third year of life with the mother. Changes in realized population growth rate between successive years were mainly driven by changes in immigration and population structure, specifically the proportional abundance of breeding females with a 2‐year‐old calf. The role of immigration as a driver of the dynamics of cetacean populations has never been quantified or considered before. Brault and Caswell (1993) used classical (i.e., not transient) LTRE and found that variance in growth rate was due to variance in adult reproductive output. Here, we also highlight the importance of dispersal through immigration processes.

## 5 CONCLUSIONS

The IPM framework here used combines information from different datasets and allows the simultaneous estimation of vital rates, including latent parameters like immigration, and population structure, while accounting for sampling and process covariance among demographic rates. Estimation of vital rates is associated with estimation of stage‐specific and total abundance and realized population growth rates, as opposed to asymptotic growth rates that require assumptions like stationary environment and a stable stage distribution. In order to exploit all available information, we made use of data for individuals that do not have permanent marks and cannot be identified across multiple years, by incorporating them into year‐specific population counts. These data were formally integrated with encounter‐reencounter data from photo‐identification of individuals with permanent marks classically used for the encounter‐reencounter analysis. With regard to the latter, our multievent model incorporates reproductive parameters following Couet et al. (2019) but could be further complicated for other species to account for variable litter size and uncertainty on the timing at offspring independence (Cubaynes et al., 2021). The model could also be extended to include spatial information, that is, spatial explicit encounter‐reencounter data, which might account for dispersal.

Our study provides insight into the immigration and reproductive processes that drive population dynamics of a poorly known cetacean. Quantifying vital rates and population structure, along with anthropogenic impacts and their population consequences are of primary concern to inform knowledge‐based conservation actions (Hooker et al., 2019; Li & Rosso, 2021). Deep‐diving species appear in fact particularly susceptible to acute effects of mid‐frequency active sonar exposure, and Cuvier's beaked whales remain the most frequently observed species in sonar‐associated stranding events worldwide (Curtis et al., 2021; Hooker et al., 2019). This poorly known, cryptic cetacean has thus become the focus of a wide range of studies that are collecting demographic data. We presented an analytical approach for maximizing the use of available data through the integration of multiple sources of information for individuals of different distinctiveness levels. By jointly using IPM estimates and tLTRE (Koons et al., 2016, 2017), we have also shown how this framework allows for more insights into the relative demographic role of different vital rates and population structure in a cetacean, while accounting for nonstationary environments.

Overall, given the large number of population studies on cetacean species that routinely record encounter‐reencounter information, the approach presented here can be applied to other ecological systems to better understand the demographic contribution of life‐history traits and population structure. This will represent a step forward to improve the assessment of how different human activities jointly influence the persistence of cetacean populations in addition to the increasingly recognized impacts on animal behavior (Nabe‐Nielsen et al., 2018).

## AUTHOR CONTRIBUTIONS

**Simone Tenan:** Conceptualization (lead); formal analysis (lead); methodology (lead); resources (lead); software (lead); writing – original draft (lead); writing – review and editing (lead). **Aurelie Moulins:** Investigation (supporting); project administration (supporting). **Paola Tepsich:** Funding acquisition (supporting); investigation (supporting); project administration (supporting). **Alessandro Bocconcelli:** Investigation (supporting). **Alessandro Verga:** Investigation (supporting). **Marco Ballardini:** Investigation (supporting). **Barbara Nani:** Investigation (supporting). **Daniela Papi:** Investigation (supporting). **Gabriella Motta:** Investigation (supporting). **Ana Sanz Aguilar:** Methodology (supporting); writing – original draft (supporting); writing – review and editing (supporting). **Massimiliano Rosso:** Conceptualization (lead); data curation (lead); funding acquisition (lead); investigation (lead); methodology (lead); project administration (lead); resources (lead); supervision (lead); writing – original draft (supporting); writing – review and editing (supporting).

## 6 FUNDING INFORMATION

None.

## Supporting information


Appendix S1.

Appendix S2.

## Data Availability

The data used in this paper are available on the Figshare Repository https://doi.org/10.6084/m9.figshare.20038226.
