# Supplementary material for: Immigration as the main driver of population dynamics in a cryptic cetacean
Source: Ecol Evol. 2023 Feb 11;13(2):e9806. doi: 10.1002/ece3.9806 (PMC9919498; doi:10.1002/ece3.9806)
Supplement: Supplementary file 1 — Appendix S1. [file ECE3-13-e9806-s002.pdf]

Simone Tenan, Aurelie Moulins, Paola Tepsich, Alessandro Bocconcelli, Alessandro Verga, Marco Ballardini, Barbara Nani, Daniela Papi, Gabriella Motta, Ana Sanz Aguilar, and Massimiliano Rosso

# Immigration as the main driver of population dynamics in a cryptic cetacean

Appendix S1: Description and code of the integrated population model, goodness-of-fit tests, transient life table response experiments, and selection of the multievent models.

## S.1 The integrated population model

### S.1.1 Population model

We used a stage-structured stochastic population model that links the demographic rates with changes in population size from year $t$ to $t+1$. Demographic stochasticity was considered by using the following distributional assumptions for females (hereafter subscript 'F' = female, 'M' = male):

$$N^{part1a}_{Juv,FM,t} \sim \text{Bin}\Big(N_{Bc2,F,t-1},\ \pi_{1,t-1}\Big) \tag{S1}$$

$$\text{with } \pi_{1,t-1} = \phi_{F,t-1}\,\phi_{By,F,t-1}\,(1-F_{t-1}) \tag{S2}$$

$$N^{part1a}_{Juv,F,t} \sim \text{Bin}\Big(N^{part1a}_{Juv,FM,t},\ 0.5\Big) \tag{S3}$$

$$N_{Bc3,F,t} \sim \text{Bin}\Big((N_{Bc2,F,t-1} - N^{part1a}_{Juv,FM,t}),\ \pi_{2,t-1}\Big) \tag{S4}$$

$$\text{with } \pi_{2,t-1} = \frac{(\phi_{F,t-1}\,\phi_{By,F,t-1}\,F_{t-1})}{(\phi_{F,t-1}\,\phi_{By,F,t-1}\,F_{t-1}) + (\phi_{F,t-1}\,(1-\phi_{By,F,t-1})) + (1-\phi_{F,t-1})} \tag{S5}$$

$$N_{De23,t} \sim \text{Bin}\Big((N_{Bc2,F,t-1} - N^{part1a}_{Juv,FM,t} - N_{Bc3,F,t}),\ \pi_{3,t-1}\Big) \tag{S6}$$

$$\text{with } \pi_{3,t-1} = \frac{(\phi_{F,t-1}\,(1-\phi_{By,F,t-1}))}{(\phi_{F,t-1}\,(1-\phi_{By,F,t-1})) + (1-\phi_{F,t-1})} \tag{S7}$$

$$N_{De,F,t} \sim \text{Bin}\Big((N_{Bc2,F,t-1} - N^{part1a}_{Juv,FM,t} - N_{Bc3,F,t} - N_{De23,t}),\ 1\Big) \tag{S8}$$

$$N_{w3,FM,t} = N_{Bc3,F,t} \tag{S9}$$

$$N_{w3,F,t} \sim \text{Bin}\Big(N_{w3,FM,t},\ 0.5\Big) \tag{S10}$$

$$N^{part1b}_{Juv,F,t} \sim \text{Bin}\Big(N_{w3,F,t-1},\ \phi_{F,t-1}\Big) \tag{S11}$$

$$N^{part2}_{Juv,F,t} \sim \text{Bin}\Big(N_{Juv,F,t-1},\ \phi_{F,t-1}\,(1-\psi_{JuvPB,F})\Big) \tag{S12}$$

$$N_{Imm,F,t} \sim \text{Bin}\Big(N_{Imm,FM,t},\ 0.5\Big) \tag{S13}$$

$$N_{Juv,F,t} = N^{part1a}_{Juv,F,t} + N^{part1b}_{Juv,F,t} + N^{part2}_{Juv,F,t} + N_{Imm,F,t} \tag{S14}$$

$$N^{part1}_{Pb,F,t} \sim \text{Bin}\Big(N_{Juv,F,t-1},\ \phi_{F,t-1}\,\psi_{JuvPB,F}\Big) \tag{S15}$$

$$N^{part2}_{Pb,F,t} \sim \text{Bin}\Big(N_{Pb,F,t-1},\ \phi_{F,t-1}\,(1-\psi_{PbBy,F})\Big) \tag{S16}$$

$$N_{Pb,F,t} = N^{part1}_{Pb,F,t} + N^{part2}_{Pb,F,t} \tag{S17}$$

$$N^{part1}_{By,F,t} \sim \text{Bin}\Big(N_{Pb,F,t-1},\ \phi_{F,t-1}\,\psi_{PbBy,F}\Big) \tag{S18}$$

$$N^{part2}_{By,F,t} \sim \text{Bin}\Big(N_{Nb,F,t-1},\ \phi_{F,t-1}\,\gamma_{t-1}\Big) \tag{S19}$$

$$N_{By,F,t} = N^{part1}_{By,F,t} + N^{part2}_{By,F,t} \tag{S20}$$

$$N_{Bc1,F,t} \sim \text{Bin}\Big(N_{By,F,t-1},\ \phi_{F,t-1}\,\phi_{By,F,t-1}\Big) \tag{S21}$$

$$N_{Bc2,F,t} \sim \text{Bin}\Big(N_{Bc1,F,t-1},\ \phi_{F,t-1}\,\phi_{By,F,t-1}\Big) \tag{S22}$$

$$N^{part1a}_{Nb,F,t} = N^{part1a}_{Juv,FM,t} \tag{S23}$$

$$N^{part1b}_{Nb,F,t} \sim \text{Bin}\Big(N_{Bc3,F,t-1},\ \phi_{F,t-1}\Big) \tag{S24}$$

$$N^{part1c}_{Nb,F,t} = N_{De23,t} \tag{S25}$$

$$N^{part1d}_{Nb,F,t} \sim \text{Bin}\Big(N_{Bc1,F,t-1},\ \phi_{F,t-1}\,(1-\phi_{By,F,t-1})\Big) \tag{S26}$$

$$N^{part1e}_{Nb,F,t} \sim \text{Bin}\Big(N_{By,F,t-1},\ \phi_{F,t-1}\,(1-\phi_{By,F,t-1})\Big) \tag{S27}$$

$$N^{part2}_{Nb,F,t} \sim \text{Bin}\Big(N_{Nb,F,t-1},\ \phi_{F,t-1}\,(1-\gamma_{t-1})\Big) \tag{S28}$$

$$N_{Nb,F,t} = N^{part1a}_{Nb,F,t} + N^{part1b}_{Nb,F,t} + N^{part1c}_{Nb,F,t} + N^{part1d}_{Nb,F,t} + N^{part1e}_{Nb,F,t} + N^{part2}_{Nb,F,t} \tag{S29}$$

where stage-specific abundance parameters are detailed in Table S1, $\phi_{F,t}$ denotes the apparent survival probability from year $t$ to $t+1$ for females (equal for all stages; see Section S.3), $\phi_{By,F,t}$ denotes the survival probability for young-of-the-year and calves from year $t$ to $t+1$ (conditional on mother survival $\phi_{F,t}$), $F_t$ indicates the probability of remaining with the mother for a calf from year $t$ to $t+1$ (conditional on survival $\phi_{By,F,t}$ and fixed to 1 for young-of-the-year and 1-year old calves). Note that in the multievent model for encounter-reencounter data (see below Section S.1.2) we modelled the apparent survival probability, $\phi_{Bc,F,t}$, for 1-year-old calves and 2-year-old calves that remain with the mother up to the third year of life. However, apparent survival probability for 2-year-old calves may be biased since true mortality cannot be distinguished from calf emancipation from its mother (Couet et al. 2019). In the state-space model we thus assumed that true survival for a 2-year old calf was equal to $\phi_{By,t}$, i.e. suvival of young-of-the-year and 1-year-old calves that cannot yet emancipate, which gives (conditional on mother survival) $\phi_{Bc,F,t} = \phi_{By,F,t}\,F_t$, and probability of remaining with the mother $F_t = \frac{\phi_{Bc,F,t}}{\phi_{By,F,t}}$.

Note that equations S1-S8 represent a sequence of binomial random variables that are conditionally related to one another (Kéry and Royle 2015) to express the vector of multinomial number of individuals originated from $N_{Bc2,F,t}$, i.e. the number of breeding females with a 2-year-old calf at year $t$ ($N^{part1a}_{Juv,FM,t}$, number of juveniles at year $t$ from

2-year-old calves weaned between $t-1$ and $t$; $N_{Bc3,F,t}$, number of breeding females with a 3-year-old calf at $t$; $N_{De23,t}$, number of calves died, from $t-1$ to $t$, between 2 and 3 years of age (mother alive); $N_{De,F,t}$, number of breeding females died, from $t-1$ to $t$, with a calf between 2 and 3 years of age) with related probabilities reported in Fig. S1:

$$\{N^{part1a}_{Juv,FM,t}, N_{Bc3,F,t}, N_{De23,t}, N_{De,F,t}\} \sim \text{Multinom}\left(N_{Bc2,F,t-1}, \boldsymbol{prob_{t-1}}\right) \quad \text{(S30)}$$

where elements of vector $\boldsymbol{prob_{t-1}}$ are: $prob_{1,t-1} = \phi_{F,t-1}\,\phi_{By,F,t-1}\,(1-F_{t-1})$ (option 3 in Fig. S1), $prob_{2,t-1} = \phi_{F,t-1}\,\phi_{By,F,t-1}\,F_{t-1}$ (option 4 in Fig. S1), $prob_{3,t-1} = \phi_{F,t-1}\,(1-\phi_{By,F,t-1})$ (option 2 in Fig. S1), $prob_{4,t-1} = (1-\phi_{F,t-1})$ (option 1 in Fig. S1).

Transition probability from juvenile to pre-breeding female is denoted by $\psi_{JuvPB,F}$, and transition probability from pre-breeding to breeding female (i.e. probability of first reproduction) is denoted by $\psi_{PbBy,F}$. Both transition probabilities are time invariant. Breeding probability for non-breeding females is indicated by $\gamma_t$.

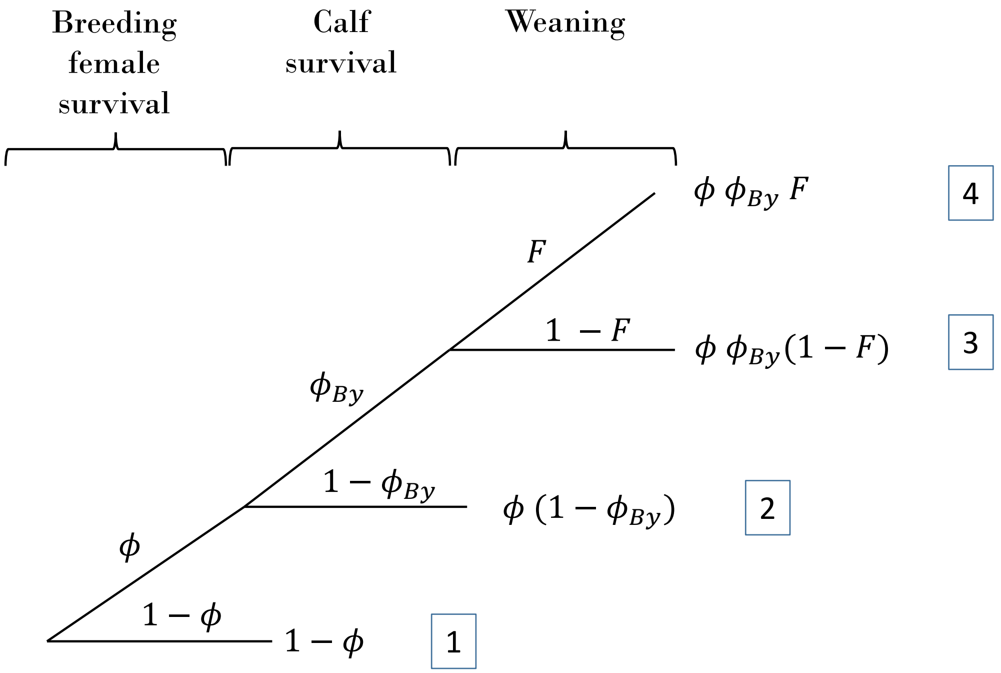


Figure S1 Graphical representation of survival and weaning processes for a breeding female and her 2- to 3-year old calf. Fate envisaged four possibilities: (1) death of breeding female (which implies the death of her calf), (2) breeding female survival but death of her calf, or (3) calf survival and weaning between 2 and 3 years old, or (4) calf survival that stay with the mother until 3 years of age. We then assumed weaning of all calves that are still with the mother at 3 years of age. Breeding female apparent survival probability is denoted by $\phi_F$, calf survival (conditional on breeding female apparent survival) is indicated by $\phi_{By,F}$, and probability of remaining with the mother is indicated by $F$.

Table S1: Notation of different population structure parameters for females.

| Parameter | significance |
|---|---|
| $N_{Juv,FM,t}^{part1a}$ | number of juveniles (females + males) at year $t$ from 2-year-old calves weaned between $t-1$ and $t$ |
| $N_{Juv,F,t}^{part1a}$ | number of juvenile females at $t$ from 2-year-old calves weaned between $t-1$ and $t$ (assuming even sex ratio) |
| $N_{Bc2,F,t}$ | number of breeding females with a 2-year-old calf at $t$ |
| $N_{Bc3,F,t}$ | number of breeding females with a 3-year-old calf at $t$ |
| $N_{De23,t}$ | number of calves died, from $t-1$ to $t$, between 2 and 3 years of age (mother alive) |
| $N_{De,F,t}$ | number of breeding females died, from $t-1$ to $t$, with a calf between 2 and 3 years of age |
| $N_{w3,FM,t}$ | number of 3-year-old calves weaned at $t$ (females + males) |
| $N_{w3,F,t}$ | number of 3-year-old female calves weaned at $t$ |
| $N_{Juv,F,t}^{part1b}$ | number of juvenile females at $t$ from 3-year-old calves weaned at $t-1$ |
| $N_{Juv,F,t}^{part2}$ | number of juvenile females at $t$ from juvenile females at $t-1$ |
| $N_{Imm,F,t}$ | number of immigrant females at $t$ (assumed juveniles) |
| $N_{Juv,F,t}$ | total number of juvenile females at $t$ |
| $N_{Pb,F,t}^{part1}$ | number of pre-breeding females at $t$ from juvenile females at $t-1$ |
| $N_{Pb,F,t}^{part2}$ | number of pre-breeding females at $t$ from pre-breeding females at $t-1$ |
| $N_{Pb,F,t}$ | total number of pre-breeding females at $t$ |
| $N_{By,F,t}^{part1}$ | number of breeding females at $t$ with a young-of-the-year, from pre-breeding females at $t-1$ |
| $N_{By,F,t}^{part2}$ | number of breeding females at $t$ with a young-of-the-year, from non-breeding females at $t-1$ |
| $N_{By,F,t}$ | total number of breeding females at $t$ with a young-of-the-year |
| $N_{Bc1,F,t}$ | number of breeding females at $t$ with a 1-year-old calf |
| $N_{Nb,F,t}^{part1a}$ | number of non-breeding females at $t$ that were breeding females at $t-1$ with calf weaned between 2 and 3 years of age |
| $N_{Nb,F,t}^{part1b}$ | number of non-breeding females at $t$ that were breeding females at $t-1$ with calf weaned at 3 years of age |
| $N_{Nb,F,t}^{part1c}$ | number of non-breeding females at $t$ that were breeding females at $t-1$ and have lost their calf between 2 and 3 years of age |
| $N_{Nb,F,t}^{part1d}$ | number of non-breeding females at $t$ that were breeding females at $t-1$ and have lost their 1-year-old calf |
| $N_{Nb,F,t}^{part1e}$ | number of non-breeding females at $t$ that were breeding females at $t-1$ and have lost their young-of-the-year |
| $N_{Nb,F,t}^{part2}$ | number of non-breeding females at $t$ that were non-breeding females at $t-1$ |
| $N_{Nb,F,t}$ | total number of non-breeding females at $t$ |

Demographic stochasticity for the male part of the population was modelled as follows:

$$N^{part1a}_{Juv,M,t} = N^{part1a}_{Juv,FM,t} - N^{part1a}_{Juv,F,t} \quad \text{(S31)}$$

$$N_{w3,M,t} = N_{w3,FM,t} - N_{w3,F,t} \quad \text{(S32)}$$

$$N^{part1b}_{Juv,M,t} \sim \text{Bin}\Big(N_{w3,M,t-1},\ \phi_{JuvSub,M,t-1}\Big) \quad \text{(S33)}$$

$$N^{part2}_{Juv,M,t} \sim \text{Bin}\Big(N_{Juv,M,t-1},\ \phi_{JuvSub,M,t-1}\,(1-\psi_M)\Big) \quad \text{(S34)}$$

$$N_{Imm,M,t} = N_{Imm,FM,t} - N_{Imm,F,t} \quad \text{(S35)}$$

$$N_{Juv,M,t} = N^{part1a}_{Juv,M,t} + N^{part1b}_{Juv,M,t} + N^{part2}_{Juv,M,t} + N_{Imm,M,t} \quad \text{(S36)}$$

$$N^{part1}_{Sub,M,t} \sim \text{Bin}\Big(N_{Juv,M,t-1},\ \phi_{JuvSub,M,t-1}\,\psi_M\Big) \quad \text{(S37)}$$

$$N^{part2}_{Sub,M,t} \sim \text{Bin}\Big(N_{Sub,M,t-1},\ \phi_{JuvSub,M,t-1}\,(1-\psi_M)\Big) \quad \text{(S38)}$$

$$N_{Sub,M,t} = N^{part1}_{Sub,M,t} + N^{part2}_{Sub,M,t} \quad \text{(S39)}$$

$$N^{part1}_{AdNt,M,t} \sim \text{Bin}\Big(N_{Sub,M,t-1},\ \phi_{JuvSub,M,t-1}\,\psi_M\Big) \quad \text{(S40)}$$

$$N^{part2}_{AdNt,M,t} \sim \text{Bin}\Big(N_{AdNt,M,t-1},\ \phi_{Ad,M,t-1}\,(1-\psi_M)\Big) \quad \text{(S41)}$$

$$N_{AdNt,M,t} = N^{part1}_{AdNt,M,t} + N^{part2}_{AdNt,M,t} \quad \text{(S42)}$$

$$N^{part1}_{Ad,M,t} \sim \text{Bin}\Big(N_{AdNt,M,t-1},\ \phi_{Ad,M,t-1}\,\psi_M\Big) \quad \text{(S43)}$$

$$N^{part2}_{Ad,M,t} \sim \text{Bin}\Big(N_{Ad,M,t-1},\ \phi_{Ad,M,t-1}\Big) \quad \text{(S44)}$$

$$N_{Ad,M,t} = N^{part1}_{Ad,M,t} + N^{part2}_{Ad,M,t}, \quad \text{(S45)}$$

where stage-specific abundance parameters are detailed in Table S2, $\phi_{JuvSub,M,t}$ denotes apparent survival probability for juvenile and subadult males from year $t$ to $t+1$, $\phi_{Ad,M,t}$ denotes apparent survival probability for adult males (both not toothed and toothed) from year $t$ to $t+1$, and $\psi_M$ indicates transition probability (equal among stages and time invariant).

Total number of immigrants (females and males) at year $t$ was derived as:

$$N_{Imm,FM,t} \sim \text{Pois}(\omega_t) \quad \text{(S46)}$$

where $\omega_t$ is the expected number of immigrants in year $t$ (Schaub et al. 2013). Immigration was modelled with random year effects:

$$\log(\omega_t) = \mu_\omega + \epsilon_{\omega,t} \text{ with } \epsilon_{\omega,t} \sim \text{Norm}(0, \sigma^2_\omega). \quad \text{(S47)}$$

The expected number of immigrants was not sex-specific to reduce bias in the estimation of this latent parameter,

Table S2: Notation of different population structure parameters for males.

| Parameter | significance |
|---|---|
| $N^{part1a}_{Juv,M,t}$ | number of juvenile males at $t$ from 2-year-old calves weaned between $t-1$ and $t$ (assuming even sex ratio) |
| $N_{w3,M,t}$ | number of 3-year-old male calves weaned at $t$ |
| $N^{part1b}_{Juv,M,t}$ | number of juvenile males at $t$ from 3-year-old calves weaned at $t-1$ |
| $N^{part2}_{Juv,M,t}$ | number of juvenile males at $t$ from juvenile males at $t-1$ |
| $N_{Imm,M,t}$ | number of immigrant males at $t$ (assumed juveniles) |
| $N_{Juv,M,t}$ | total number of juvenile males at $t$ |
| $N^{part1}_{Sub,M,t}$ | number of subadult males at $t$ from juvenile males at $t-1$ |
| $N^{part2}_{Sub,M,t}$ | number of subadult males at $t$ from subadult males at $t-1$ |
| $N_{Sub,M,t}$ | total number of subadult males at $t$ |
| $N^{part1}_{AdNt,M,t}$ | number of not toothed adult males at $t$ from subadult males at $t-1$ |
| $N^{part2}_{AdNt,M,t}$ | number of not toothed adult males at $t$ from not toothed adult males at $t-1$ |
| $N_{AdNt,M,t}$ | total number of not toothed adult males at $t$ |
| $N^{part1}_{Ad,M,t}$ | number of toothed adult males at $t$ from not toothed males at $t-1$ |
| $N^{part2}_{Ad,M,t}$ | number of toothed adult males at $t$ from toothed males at $t-1$ |
| $N_{Ad,M,t}$ | total number of toothed adult males at $t$ |

assuming even sex ratio among immigrants (Eqs. S13 and S35). In addition, immigration was assumed to occur in the juvenile stage.

Number of individuals in eqs. S1-29 and S31-S45 were used to derive the following quantities:

$$N_{Juv,FM,t} = N_{Juv,F,t} + N_{Juv,M,t} \tag{S48}$$

$$N_{Nr,FM,t} = N_{Pb,F,t} + N_{Nb,F,t} + N_{Sub,M,t} + N_{AdNt,M,t} \tag{S49}$$

$$N_{Br,F,t} = N_{By,F,t} + N_{Bc1,F,t} + N_{Bc2,F,t} + N_{Bc3,F,t} \tag{S50}$$

$$N_{Tot,F,t} = N_{Juv,F,t} + N_{Pb,F,t} + N_{Br,F,t} + N_{Nb,F,t} \tag{S51}$$

$$N_{Tot,M,t} = N_{Juv,M,t} + N_{Sub,M,t} + N_{AdNt,M,t} + N_{Ad,M,t} \tag{S52}$$

$$N_{Tot,FM,t} = N_{Tot,F,t} + N_{Tot,M,t} \tag{S53}$$

where $N_{Juv,FM,t}$ is the total number of juveniles in the population at year $t$, $N_{Nr,FM,t}$ is the total number of non-reproductive individuals at $t$ (i.e. immatures that have not yet recruited as breeders, excluding juveniles, and non-breeders that have reproduced at least once in the past), $N_{Br,F,t}$ is the total number of breeding females at $t$,

$N_{Tot,F,t}$ is the total number of females at $t$, $N_{Tot,M,t}$ is the total number of males at $t$, and $N_{Tot,FM,t}$ is the total number of individuals in the population at $t$.

In the observation (sub)models we described the relationship between counts of stage-specific number of individuals ($y_t$) and true population size as:

$$y_{Juv,FM,t} \sim \text{Bin}\Big(N_{Juv,FM,t},\ p_{Juv,FM,t}\Big) \tag{S54}$$

$$y_{Nr,FM,t} \sim \text{Bin}\Big(N_{Nr,FM,t},\ p_{Nr,FM,t}\Big) \tag{S55}$$

$$y_{Br,F,t} \sim \text{Bin}\Big(N_{Br,F,t},\ p_{F,t}\Big) \tag{S56}$$

$$y_{Br,M,t} \sim \text{Bin}\Big(N_{Ad,M,t},\ p_{Ad,M,t}\Big) \tag{S57}$$

where $y_{Juv,FM,t}$, $y_{Nr,FM,t}$, $y_{Br,F,t}$, and $y_{Br,M,t}$ denoted counts of juveniles, non-reproductive individuals (i.e. immatures that have not yet recruited as breeders, excluding juveniles, and non-breeders that have reproduced at least once in the past), breeding females, and adult toothed males at year $t$, respectively. Given the observation process for count data was the same as for photo-identification encounter-reencounter data, the observation models share the sex- and/or stage-specific detection probability parameters with the multievent model for encounter-reencounter data (see Section S.1.2). Specifically, detection probability for juveniels (females and males) was the average detectability for juveniles of the two sexes, $p_{Juv,FM,t} = (p_{F,t} + p_{Juv,M,t})/2$ (eqs. S59 and S61 for the multievent model), detection probability for non-reproductive individuals was the average of the related sex- and stage-specific probabilities $p_{Nr,FM,t} = (p_{F,t} + p_{Sub,M,t} + p_{AdNt,M,t})/3$ (eqs. S59 and S61), whereas detection probability for breeding females ($p_{F,t}$) and for adult toothed males ($p_{Ad,M,t}$) were shared directly with the multievent model (eqs. S59 and S61).

Annual sex- and/or stage-specific counts of the total number of individuals observed in the population in 2004-2019 were obtained by considering photo-identification encounter-reencounter data of all individuals identified with permanent and ephemeral marks (distinctiveness levels 1 and 2). Note that for the multievent model only encounter-reencounter data of individuals with permanent marks (distinctiveness level 2) were considered. In addition, given the presence of juveniles and non-reproductive individuals with unknown sex, for these two groups counts we pulled all count data together for females, males and unknowns, in order to exploit all available data. (Table S3-S4).

Table S3: Total number of individuals counted in the population during the period 2004-2019 for each sex and category. The non-reproductive category includes immatures that have not yet recruited as breeders, excluding juveniles, and non-breeders that have reproduced at least once in the past. The reproductive category includes breeding females and adult toothed males. The proportion of counted individuals with unknown sex is also reported. Note that the sum of the figures here reported (254) does not correspond to the total number of unique individuals counted (250) since some individuals were present in counts as juvenile or non-reproductive with unknown sex but then resighted as reproductive individuals with known sex.

| Sex | Juvenile | Non-reproductive | Reproductive |
|---|---|---|---|
| Female | 9 | 32 | 28 |
| Male | 22 | 56 | 45 |
| Unknown | 53 | 20 | 0 |
| % Unknown | 0.64 | 0.18 | 0 |

Table S4: Year-specific number of individuals counted in the population, during the period 2004-2019, for each sex and category. The non-reproductive category includes immatures that have not yet recruited as breeders, excluding juveniles, and non-breeders that have reproduced at least once in the past. The reproductive category includes breeding females and adult toothed males.

| | Juvenile | | | | Non-reproductive | | | | Reproductive | |
|---|---|---|---|---|---|---|---|---|---|---|
| | Female | Male | Unknown | Total | Female | Male | Unknown | Total | Female | Male |
| 2004 | 2 | 3 | 1 | 6 | 2 | 15 | 2 | 19 | 2 | 7 |
| 2005 | 4 | 5 | 3 | 12 | 3 | 13 | 3 | 19 | 15 | 24 |
| 2006 | 5 | 6 | 6 | 17 | 4 | 13 | 6 | 23 | 5 | 11 |
| 2007 | 1 | 1 | 0 | 2 | 1 | 4 | 1 | 6 | 3 | 3 |
| 2008 | 1 | 1 | 0 | 2 | 0 | 2 | 0 | 2 | 4 | 2 |
| 2009 | 0 | 1 | 0 | 1 | 3 | 2 | 1 | 6 | 1 | 5 |
| 2010 | 0 | 0 | 1 | 1 | 0 | 1 | 0 | 1 | 1 | 0 |
| 2011 | 0 | 2 | 3 | 5 | 0 | 4 | 3 | 7 | 1 | 9 |
| 2012 | 0 | 3 | 2 | 5 | 2 | 5 | 0 | 7 | 4 | 8 |
| 2013 | 0 | 3 | 8 | 11 | 8 | 14 | 1 | 23 | 5 | 12 |
| 2014 | 0 | 3 | 8 | 11 | 5 | 8 | 3 | 16 | 9 | 19 |
| 2015 | 0 | 4 | 11 | 15 | 6 | 18 | 4 | 28 | 11 | 20 |
| 2016 | 1 | 2 | 7 | 10 | 5 | 11 | 2 | 18 | 6 | 13 |
| 2017 | 0 | 0 | 10 | 10 | 2 | 18 | 3 | 23 | 12 | 20 |
| 2018 | 0 | 1 | 16 | 17 | 7 | 16 | 8 | 31 | 16 | 28 |
| 2019 | 0 | 0 | 11 | 11 | 3 | 15 | 9 | 27 | 16 | 20 |

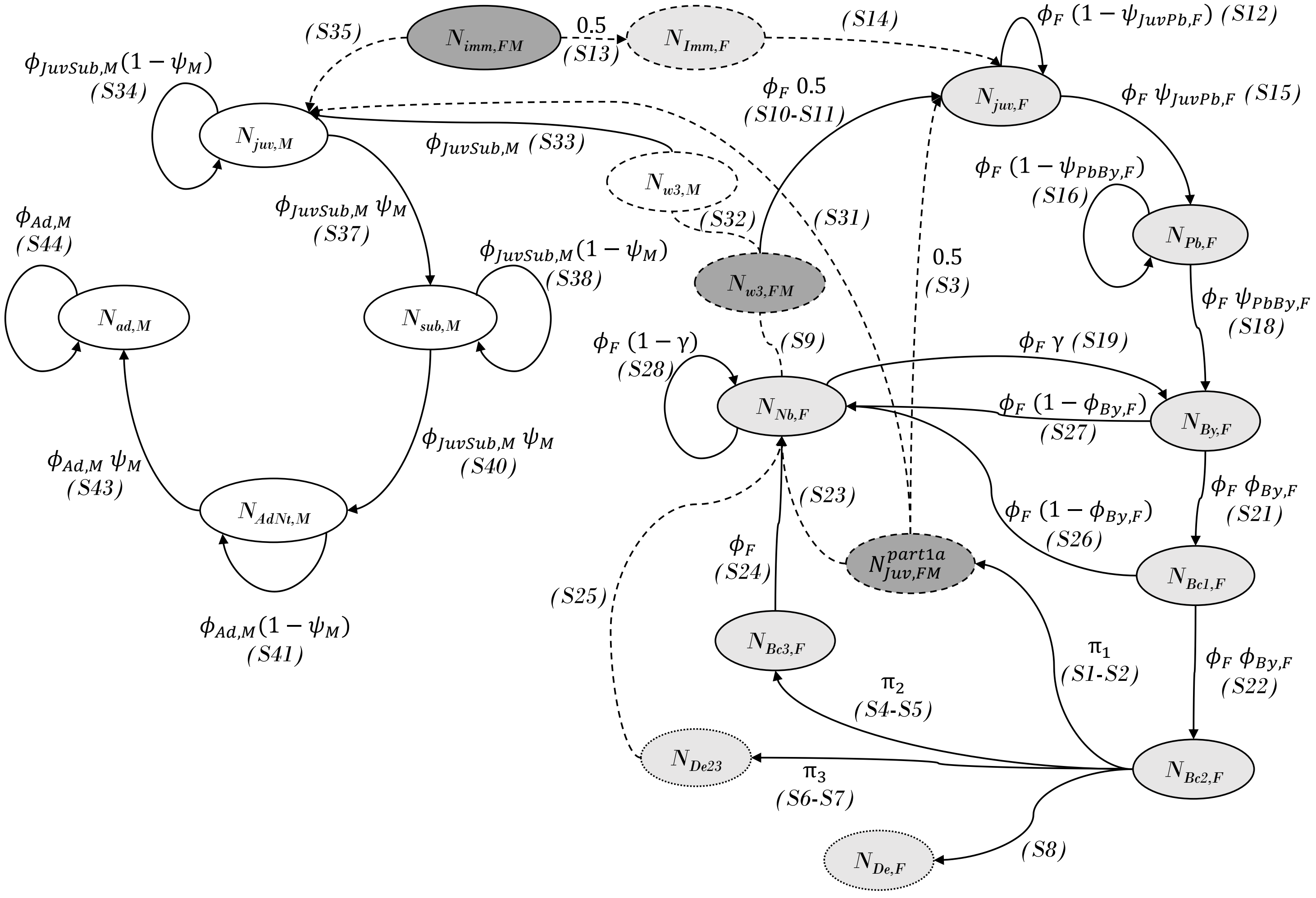


Figure S2 Annual life cycle for the Cuvier's beaked whale model. Population stages for males (white background), females (light gray) or both sexes (dark grey) are reported following equations (number in parentheses) of the population model. Intermediate (i.e. within year) stages and transitions are indicated by dotted circles and arrows. Stages are defined in Table S1 and S2.

### S.1.2 Individual encounter histories

We used sex-specific multievent capture-recapture models (Pradel 2005) for the individual photo-identification encounter-reencounter data information. Multievent models are capture-recapture models able to accommodate uncertainty in state assignment and to model multiple state transitions and observational processes (Pradel 2005). We considered eight biological states for females: 'juvenile' ($Juv$), 'pre-breeder' ($Pb$), 'breeder with a young-of-the-year' ($By$), 'breeder with a 1-year-old calf' ($Bc1$), 'breeder with a 2-year-old calf' ($Bc2$), 'breeder with a 3-year-old calf' ($Bc3$), 'non-breeder' with previous breeding experience ($Nb$), and 'dead' ($D$). Individual $i$ can change state ($z_{i,t}$) according to transition matrix $\mathbf{\Delta}_F$ (eq. S58) with a state equation $\boldsymbol{z}_{F,i,t+1}|\boldsymbol{z}_{F,i,t} \sim \text{Cat}(\boldsymbol{z}_{F,i,t}\,\mathbf{\Delta}_{F,i,t})$. Seven types of mutually exclusive events ($x_{i,t}$) could be observed and were arbitrarily coded as follows: '1' = 'not seen', '2' = 'seen as juvenile', '3' = 'seen as pre-breeder', '4' = 'seen as breeder with a young-of-the-year', '5' = 'seen as breeder with a 1-year-old or older calf', '6' = 'seen as non-breeder', '7' = 'seen alone'. Event '7' could include either a pre-breeder or a non-breeder, that may be difficult to distinguish based on colouring. We assumed that all breeding states were ascertained with certainty. The observation equation was $\boldsymbol{x}_{F,i,t}|z_{F,i,t} \sim \text{Cat}(\boldsymbol{x}_{F,i,t}\,\mathbf{\Theta}_{F,i,t})$, with $\mathbf{\Theta}_{F,i,t}$ denoting the observation matrix (eq. S59). The latter includes female encounter (resighting) probability ($p_{F,t}$) and the (time-invariant) probability that the state of the individual was ascertained during observation ($\delta_F$, equal for pre-breeding and non-breeding females).

We assumed five states for males: 'juvenile' ($Juv$), 'subadult' ($Sub$), 'not toothed adult male' ($AdNt$), 'toothed adult male' ($Ad$), and 'dead' ($D$). As for females, individual $i$ can change state ($z_{i,t}$) according to transition matrix $\mathbf{\Delta}_M$ (eq. S60) with a state equation $z_{M,i,t+1}|z_{M,i,t} \sim \text{Cat}(z_{M,i,t}\,\mathbf{\Delta}_{M,i,t})$. Six types of mutually exclusive events ($x_{i,t}$) could be observed and were arbitrarily coded as follows: '1' = 'not seen', '2' = 'seen as juvenile', '3' = 'seen as subadult', '4' = 'seen as not toothed adult', '5' = 'seen as toothed adult', '6' = 'seen as adult'. Event '6' could include either a not toothed or a toothed adult, being this feature not always ascertained. We assumed that all other states were ascertained with certainty based on colouring. The observation equation was $\boldsymbol{x}_{M,i,t}|z_{M,i,t} \sim \text{Cat}(\boldsymbol{x}_{M,i,t}\,\mathbf{\Theta}_{M,i,t})$, with $\mathbf{\Theta}_{M,i,t}$ denoting the observation matrix (eq. S61). The latter includes male encounter (resighting) probability for juveniles ($p_{Juv,M,t}$), subadults ($p_{Sub,M,t}$), not toothed adults ($p_{AdNt,M,t}$), toothed adults ($p_{Ad,M,t}$), and the (time-invariant) probabilities that the state of the individual was ascertained during observation, for not toothed and toothed adult males ($\delta_{AdNt,M}$ and $\delta_{Ad,M}$, respectively).

Multievent model structure for the two sexes, embedded in the integrated population model (IPM), was derived for each sex from the selection of a best model among a set of candidate models (see Section S.3). The selected model was then extended in the IPM by adding temporal random variation to time-variant parameters and a correlation structure among vital rates (see below).

$$
\boldsymbol{\Delta}_{F,i,t} = \begin{array}{c} \\ Juv \\ Pb \\ By \\ Bc1 \\ Bc2 \\ Bc3 \\ Nb \\ D \end{array}
\begin{array}{c}
\begin{array}{cccccccc} Juv & Pb & By & Bc1 & Bc2 & Bc3 & Nb & D \end{array} \\
\begin{pmatrix}
\phi_{F,t}\,(1-\psi_{JuvPB,F}) & \phi_{F,t}\,\psi_{JuvPB,F} & 0 & 0 & 0 & 0 & 0 & 1-\phi_{F,t} \\
0 & \phi_{F,t}\,(1-\psi_{PbBy,F}) & \phi_{F,t}\,\psi_{PbBy,F} & 0 & 0 & 0 & 0 & 1-\phi_{F,t} \\
0 & 0 & 0 & \phi_{F,t}\,\phi_{By,F,t} & 0 & 0 & \phi_{F,t}\,(1-\phi_{By,F,t}) & 1-\phi_{F,t} \\
0 & 0 & 0 & 0 & \phi_{F,t}\,\phi_{Bc,F,t} & 0 & \phi_{F,t}\,(1-\phi_{Bc,F,t}) & 1-\phi_{F,t} \\
0 & 0 & 0 & 0 & 0 & \phi_{F,t}\,\phi_{Bc,F,t} & \phi_{F,t}\,(1-\phi_{Bc,F,t}) & 1-\phi_{F,t} \\
0 & 0 & 0 & 0 & 0 & 0 & \phi_{F,t} & 1-\phi_{F,t} \\
0 & 0 & \phi_{F,t}\,\gamma_t & 0 & 0 & 0 & \phi_{F,t}\,(1-\gamma_t) & 1-\phi_{F,t} \\
0 & 0 & 0 & 0 & 0 & 0 & 0 & 1
\end{pmatrix}
\end{array}
\tag{S58}
$$

$$
\boldsymbol{\Theta}_{F,i,t} = \begin{array}{c} \\ Juv \\ Pb \\ By \\ Bc1 \\ Bc2 \\ Bc3 \\ Nb \\ D \end{array}
\begin{array}{c}
\begin{array}{ccccccc} 1 & 2 & 3 & 4 & 5 & 6 & 7 \end{array} \\
\begin{pmatrix}
(1-p_{F,t}) & p_{F,t} & 0 & 0 & 0 & 0 & 0 \\
(1-p_{F,t}) & 0 & p_{F,t}\,\delta_F & 0 & 0 & 0 & p_t\,(1-\delta_F) \\
(1-p_{F,t}) & 0 & 0 & p_{F,t} & 0 & 0 & 0 \\
(1-p_{F,t}) & 0 & 0 & 0 & p_{F,t} & 0 & 0 \\
(1-p_{F,t}) & 0 & 0 & 0 & p_{F,t} & 0 & 0 \\
(1-p_{F,t}) & 0 & 0 & 0 & p_{F,t} & 0 & 0 \\
(1-p_{F,t}) & 0 & 0 & 0 & 0 & p_{F,t}\,\delta_F & p_{F,t}\,(1-\delta_F) \\
1 & 0 & 0 & 0 & 0 & 0 & 0
\end{pmatrix}
\end{array}
\tag{S59}
$$

$$\boldsymbol{\Delta}_{M,i,t} = \begin{array}{c} \\ Juv \\ Sub \\ AdNt \\ Ad \\ D \end{array} \begin{array}{c} \begin{array}{ccccc} Juv & Sub & AdNt & Ad & D \end{array} \\ \begin{pmatrix} \phi_{JuvSub,M,t}(1-\psi_M) & \phi_{JuvSub,M,t}\,\psi_M & 0 & 0 & (1-\phi_{JuvSub,M,t}) \\ 0 & \phi_{JuvSub,M,t}(1-\psi_M) & \phi_{JuvSub,M,t}\,\psi_M & 0 & (1-\phi_{JuvSub,M,t}) \\ 0 & 0 & \phi_{Ad,M,t}(1-\psi_M) & \phi_{Ad,M,t}\,\psi_M & (1-\phi_{Ad,M,t}) \\ 0 & 0 & 0 & \phi_{Ad,M,t} & (1-\phi_{Ad,M,t}) \\ 0 & 0 & 0 & 0 & 1 \end{pmatrix} \end{array} \quad \text{(S60)}$$

$$\boldsymbol{\Theta}_{M,i,t} = \begin{array}{c} \\ Juv \\ Sub \\ AdNt \\ Ad \\ D \end{array} \begin{array}{c} \begin{array}{cccccc} 1 & 2 & 3 & 4 & 5 & 6 \end{array} \\ \begin{pmatrix} (1-p_{Juv,M,t}) & p_{Juv,M,t} & 0 & 0 & 0 & 0 \\ (1-p_{Sub,M,t}) & 0 & p_{Sub,M,t} & 0 & 0 & 0 \\ (1-p_{AdNt,M,t}) & 0 & 0 & p_{AdNt,M,t}\,\delta_{AdNt,M} & 0 & p_{AdNt,M,t}(1-\delta_{AdNt,M}) \\ (1-p_{Ad,M,t}) & 0 & 0 & 0 & p_{Ad,M,t}\,\delta_{Ad,M} & p_{Ad,M,t}(1-\delta_{Ad,M}) \\ 1 & 0 & 0 & 0 & 0 & 0 \end{pmatrix} \end{array} \quad \text{(S61)}$$

Probabilities of survival for females ($\phi_F$), young-of-the-year ($\phi_{By,F}$) and calves ($\phi_{Bc,F}$), as well as breeding probability ($\gamma$) where modelled with random year effects:

$$\text{logit}(\phi_{F,t}) = \mu_{\phi_F} + \epsilon_{\phi_F,t} \quad \text{with } \epsilon_{\phi_F,t} \sim \text{Norm}(0, \sigma^2_{\phi_F}) \quad \text{(S62)}$$

$$\text{logit}(\phi_{By,F,t}) = \mu_{\phi_{By,F}} + \epsilon_{\phi_{By,F},t} \quad \text{with } \epsilon_{\phi_{By,F},t} \sim \text{Norm}(0, \sigma^2_{\phi_{By,F}}) \quad \text{(S63)}$$

$$\text{logit}(\phi_{Bc,F,t}) = \mu_{\phi_{Bc,F}} + \epsilon_{\phi_{Bc,F},t} \quad \text{with } \epsilon_{\phi_{Bc,F},t} \sim \text{Norm}(0, \sigma^2_{\phi_{Bc,F}}) \quad \text{(S64)}$$

$$\text{logit}(\gamma_t) = \mu_\gamma + \epsilon_{\gamma,t} \quad \text{with } \epsilon_{\gamma,t} \sim \text{Norm}(0, \sigma^2_\gamma) \quad \text{(S65)}$$

with matrix $\boldsymbol{\epsilon}_F$ of the residuals assumed drawn from a multivariate normal distribution with zero mean, $\boldsymbol{\epsilon}_F \sim MVN(0, \boldsymbol{\Sigma}_F)$, and the variance-covariance matrix $\boldsymbol{\Sigma}_F$ as follows:

$$\boldsymbol{\Sigma}_F = \begin{pmatrix} \sigma^2_{\phi_F} & \rho_1\sigma_{\phi_F}\sigma_{\phi_{By}} & \rho_2\sigma_{\phi_F}\sigma_{\phi_{Bc}} & \rho_3\sigma_{\phi_F}\sigma_\gamma \\ \rho_1\sigma_{\phi_{By}}\sigma_{\phi_F} & \sigma^2_{\phi_{By}} & \rho_4\sigma_{\phi_{By}}\sigma_{\phi_{Bc}} & \rho_5\sigma_{\phi_{By}}\sigma_\gamma \\ \rho_2\sigma_{\phi_{Bc}}\sigma_{\phi_F} & \rho_4\sigma_{\phi_{Bc}}\sigma_{\phi_{By}} & \sigma^2_{\phi_{Bc}} & \rho_6\sigma_{\phi_{Bc}}\sigma_\gamma \\ \rho_3\sigma_\gamma\sigma_{\phi_F} & \rho_5\sigma_\gamma\sigma_{\phi_{By}} & \rho_6\sigma_\gamma\sigma_{\phi_{Bc}} & \sigma^2_\gamma \end{pmatrix} \quad \text{(S66)}$$

where $\rho$ is the temporal correlation between two parameters. Following (Alvarez et al. 2014), we specified a scaled inverse Wishart prior for the elements of the variance-covariance matrix $\boldsymbol{\Sigma}_F$.

Male survival probabilities where also modelled with random year effects:

$$\text{logit}(\phi_{JuvSub,\,M,\,t}) = \mu_{\phi_{JuvSub,\,M}} + \epsilon_{\phi_{JuvSub,\,M},\,t} \text{ with } \epsilon_{\phi_{JuvSub,\,M},\,t} \sim \text{Norm}(0, \sigma^2_{\phi_{JuvSub,\,M}}) \tag{S67}$$

$$\text{logit}(\phi_{Ad,\,M,\,t}) = \mu_{\phi_{Ad,\,M}} + \epsilon_{\phi_{Ad,\,M},\,t} \text{ with } \epsilon_{\phi_{Ad,\,M},\,t} \sim \text{Norm}(0, \sigma^2_{\phi_{Ad,\,M}}) \tag{S68}$$

with matrix $\boldsymbol{\epsilon}_M$ of the residuals assumed drawn from a multivariate normal distribution with zero mean, $\boldsymbol{\epsilon}_M \sim MVN(0, \boldsymbol{\Sigma}_M)$, and the variance-covariance matrix $\boldsymbol{\Sigma}_M$ as follows:

$$\boldsymbol{\Sigma}_M = \begin{pmatrix} \sigma^2_{\phi_{JuvSub,\,M}} & \rho_7 \sigma_{\phi_{JuvSub,\,M}} \sigma_{\phi_{Ad,\,M}} \\ \rho_7 \sigma_{\phi_{Ad,\,M}} \sigma_{\phi_{JuvSub,\,M}} & \sigma^2_{\phi_{Ad,\,M}} \end{pmatrix} \tag{S69}$$

where $\rho$ is the temporal correlation between the two parameters, and a scaled inverse Wishart prior was specified for the elements of the variance-covariance matrix $\boldsymbol{\Sigma}_M$ (Alvarez et al. 2014).

Temporal random effects were also used for modelling encounter probabilities of females and males, in addition to the effect of year-specific sampling effort in both sexes and a stage-specific (*Juv*, *Sub*, *AdNt*, *Ad*) effect on male probabilities (with $\text{stage}_{Juv}$ as reference level):

$$\text{logit}(p_{F,\,t}) = \mu_{p_F} + \beta_{p_F} \,\text{effort}_t + \epsilon_{p_F,\,t} \text{ with } \epsilon_{p_F,\,t} \sim \text{Norm}(0, \sigma^2_{p_F}) \tag{S70}$$

$$\text{logit}(p_{\text{stage},\,M,\,t}) = \mu_{p_M} + \text{stage} + \beta_{p_M} \,\text{effort}_t + \epsilon_{p_M,\,t} \text{ with } \epsilon_{p_M,\,t} \sim \text{Norm}(0, \sigma^2_{p_M}). \tag{S71}$$

### S.1.3 Derived parameters

#### *S.1.3.1 Population growth rate*

Population growth rate ($\lambda_t$) was calculated as the ratio of the total number of individuals (males and females) of two consecutive years, $N_{Tot,\,FM,\,t+1}/N_{Tot,\,FM,\,t}$, along with the geometric mean of all year-specific values. Female population growth rate ($\lambda_{F,\,t} = N_{Tot,\,F,\,t+1}/N_{Tot,\,F,\,t}$) was used with the transient life table response experiments (Section S.2).

#### *S.1.3.2 Immigration rate*

Immigration rate was calculated for the whole population (females and males, $\iota_{FM,\,t}$) and for each sex ($\iota_{F,\,t}$ and $\iota_{M,\,t}$ for females and males, respectively) as the number of immigrants in a specific year divided by the total number of

individuals in the previous year:

$$\iota_{FM,t} = N_{Imm,FM,t+1}/N_{Tot,FM,t} \tag{S72}$$

$$\iota_{F,t} = N_{Imm,F,t+1}/N_{Tot,F,t} \tag{S73}$$

$$\iota_{M,t} = N_{Imm,M,t+1}/N_{Tot,M,t}. \tag{S74}$$

### S.1.4 Likelihood of the integrated population model

Under the assumption of independence among the data sets, the joint likelihood of the integrated model is the product of the likelihoods of all different components. Part of the individuals were included in more than one data set, thereby violating to some extent the assumption of independence between different likelihoods. However, simulation studies showed that the violation of this assumption only has a minimal impact on parameter estimates and their related precision (Abadi et al. 2010; Weegman et al. 2020).

### S.1.5 Goodness-of-fit tests

We evaluated the goodness of fit of single submodels separately. For the state-space submodel (SSM), we used posterior predictive checks (Gelman et al. 1996) with the Freeman-Tukey statistic (Brooks et al. 2000) as an omnibus discrepancy measure for each category of individuals counted (eqs. S54-S57): $D_{ft} = \sum_t(\sqrt{y_t} - \sqrt{E(y_t)})^2$, where $y_t$ are the observed counts, and $E(y_t)$ the expected counts. Observed counts of juveniles, non-reproductive individuals (i.e. immatures that have not yet recruited as breeders, excluding juveniles, and non-breeders that have reproduced at least once in the past), breeding females, and adult toothed males at year $t$ are indicated by $y_{Juv,FM,t}$, $y_{Nr,FM,t}$, $y_{Br,F,t}$, and $y_{Br,M,t}$ (eqs. S54-S57). We also used the number of times the observed time series switched from short-terms ups and downs as an additional targeted test for the SSM (Gelman and Hill 2006).

Bayesian $P$-values for the discrepancy measures indicate an appropriate fit (i.e. Bayesian $P$-value far away from 0 and 1) for counts of all categories but the non-reproductive individuals for which fit was poorer, despite the targeted tests (i.e. number of switches and proportion of zeros) indicate no lack of fit for all categories of counts Fig. S3. For the evaluation of goodness of fit of the multievent submodel see Section S.3.

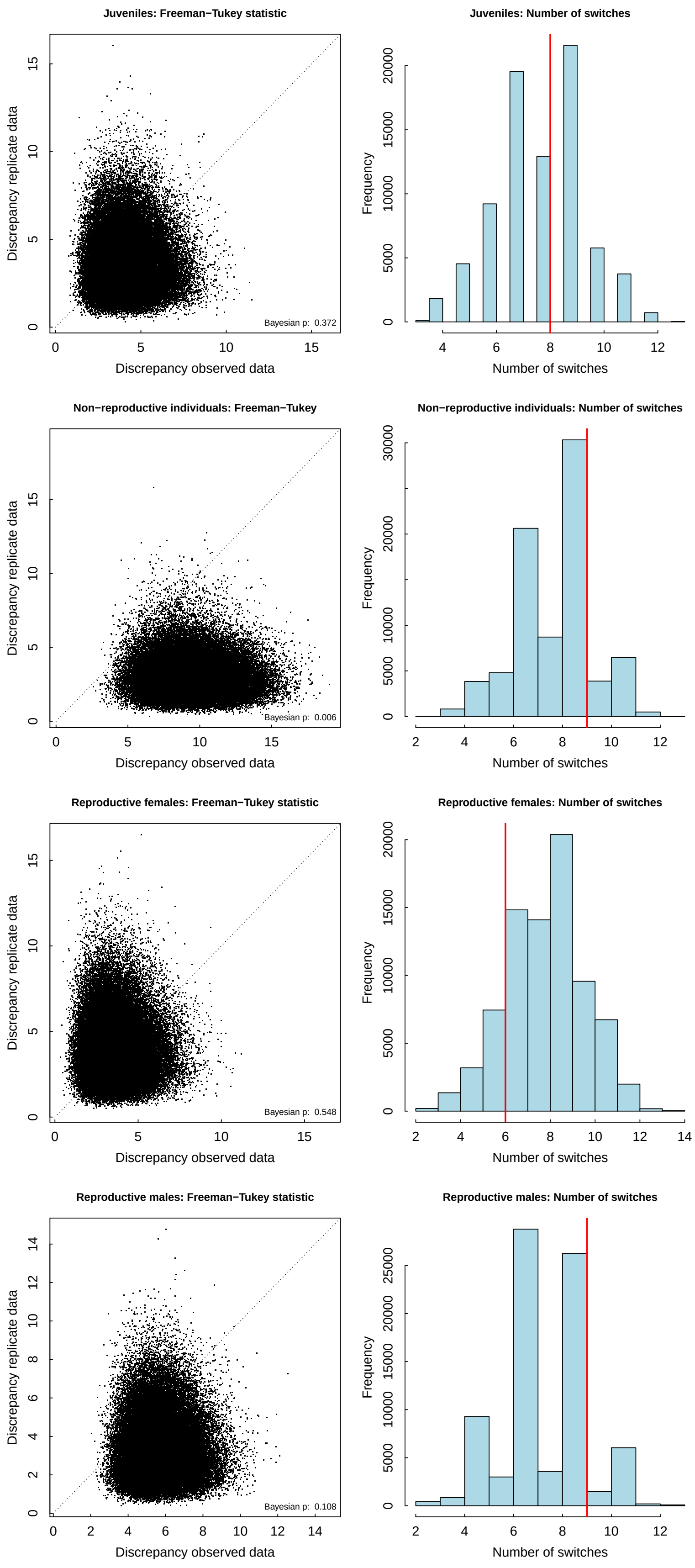


Figure S3 Graphical representation of the discrepancy measure for replicate and observed data for the state-space submodel in the integrated population model (IPM) (dot plots) and of test statistics of replicate data of a correctly specified IPM (histograms). The y=x line is shown by a dashed black line. The vertical red lines indicates test statistics of the observed data.

Table S5: Total number of individuals observed during each year, included in the encounter-reencounter dataset (i.e. individuals with permanent marks only, distinctiveness level 2).

| year | females | males | total |
|---|---|---|---|
| 2004 | 5 | 22 | 27 |
| 2005 | 13 | 30 | 43 |
| 2006 | 13 | 31 | 44 |
| 2007 | 5 | 8 | 13 |
| 2008 | 5 | 5 | 10 |
| 2009 | 4 | 7 | 11 |
| 2010 | 1 | 1 | 2 |
| 2011 | 1 | 13 | 14 |
| 2012 | 4 | 15 | 19 |
| 2013 | 12 | 30 | 42 |
| 2014 | 14 | 26 | 40 |
| 2015 | 10 | 30 | 40 |
| 2016 | 9 | 18 | 27 |
| 2017 | 10 | 30 | 40 |
| 2018 | 16 | 32 | 48 |
| 2019 | 15 | 31 | 46 |

Table S6: Total number of females observed during each year and assigned to one of the six types of mutually exclusive events*, included in the encounter-reencounter dataset (i.e. individuals with permanent marks only, distinctiveness level 2).

| year | 2 | 3 | 4 | 5 | 6 | 7 |
|---|---|---|---|---|---|---|
| 2004 | 2 | 0 | 2 | 0 | 1 | 0 |
| 2005 | 4 | 0 | 2 | 4 | 0 | 3 |
| 2006 | 4 | 0 | 0 | 1 | 4 | 4 |
| 2007 | 1 | 1 | 1 | 1 | 1 | 0 |
| 2008 | 1 | 0 | 0 | 2 | 2 | 0 |
| 2009 | 0 | 1 | 1 | 0 | 0 | 2 |
| 2010 | 0 | 0 | 0 | 0 | 1 | 0 |
| 2011 | 0 | 0 | 0 | 1 | 0 | 0 |
| 2012 | 0 | 0 | 1 | 1 | 0 | 2 |
| 2013 | 0 | 3 | 2 | 3 | 0 | 4 |
| 2014 | 0 | 3 | 0 | 4 | 4 | 3 |
| 2015 | 0 | 3 | 1 | 3 | 0 | 3 |
| 2016 | 1 | 0 | 2 | 3 | 0 | 3 |
| 2017 | 0 | 0 | 0 | 6 | 2 | 2 |
| 2018 | 0 | 1 | 1 | 3 | 5 | 6 |
| 2019 | 0 | 1 | 2 | 2 | 7 | 2 |

*Female events were arbitrarily coded as follows: '2' = 'seen as juvenile', '3' = 'seen as pre-breeder', '4' = 'seen as breeder with a young-of-the-year', '5' = 'seen as breeder with a 1-year-old or older calf', '6' = 'seen as non-breeder', '7' = 'seen alone'. Event code '1' = 'not seen' is not reported here.

Table S7: Total number of males observed during each year and assigned to one of the five types of mutually exclusive events*, included in the encounter-reencounter dataset (i.e. individuals with permanent marks only, distinctiveness level 2).

| year | 2 | 3 | 4 | 5 | 6 |
|---|---|---|---|---|---|
| 2004 | 3 | 6 | 6 | 5 | 2 |
| 2005 | 5 | 6 | 4 | 14 | 1 |
| 2006 | 7 | 7 | 5 | 9 | 3 |
| 2007 | 1 | 3 | 1 | 3 | 0 |
| 2008 | 1 | 1 | 1 | 2 | 0 |
| 2009 | 1 | 0 | 1 | 4 | 1 |
| 2010 | 0 | 1 | 0 | 0 | 0 |
| 2011 | 2 | 2 | 1 | 7 | 1 |
| 2012 | 3 | 3 | 2 | 7 | 0 |
| 2013 | 4 | 10 | 2 | 12 | 2 |
| 2014 | 3 | 7 | 2 | 13 | 1 |
| 2015 | 3 | 13 | 4 | 8 | 2 |
| 2016 | 1 | 7 | 2 | 6 | 2 |
| 2017 | 0 | 8 | 8 | 12 | 2 |
| 2018 | 1 | 6 | 8 | 13 | 4 |
| 2019 | 0 | 5 | 4 | 15 | 7 |

*Male events were arbitrarily coded as follows: '2' = 'seen as juvenile', '3' = 'seen as subadult', '4' = 'seen as not toothed adult', '5' = 'seen as toothed adult', '6' = 'seen as adult'. Event code '1' = 'not seen' is not reported here.

## S.2 Demographic influence on population growth rate: transient life table response experiments

Following Koons et al. (2016) and Koons et al. (2017), we performed a retrospective analysis through transient life table response experiments (tLTRE) using demographic rates and population structure of both sexes. Population growth rate can be expressed as $\lambda_t = N_{t+1}/N_t = \|\boldsymbol{N_{t+1}}\|/\|\boldsymbol{N_t}\| = \|\boldsymbol{A_t N_t}\|/\|\boldsymbol{N_t}\|$, where $\| \; \|$ denotes the sum of absolute values of vector elements (i.e. the 1-norm), $\boldsymbol{A_t}$ is the Leslie matrix and $\boldsymbol{N_t}$ is the population vector in year $t$. In our case study (eqs. S1-S29, S31-S45, S48-S53), we can express the product of the Leslie matrix and the population vector as:

$$\begin{aligned}
\|\boldsymbol{A_t N_t}\| = & N_{Bc2,\,F,\,t}\,\phi_{F,\,t}\,\phi_{By,F,\,t}\,(1-F_t)\,0.5 + N_{Bc3,\,F,\,t}\,\phi_{F,\,t}\,0.5 + N_{Juv,\,F,\,t}\,\phi_{F,\,t}\,(1-\psi_{JuvPB,\,F}) + \\
& N_{Juv,\,F,\,t-1}\,\phi_{F,\,t-1}\,\psi_{JuvPB,\,F} + N_{Pb,\,F,\,t}\,\phi_{F,\,t}\,(1-\psi_{PbBy,\,F}) + \\
& N_{Pb,\,F,\,t}\,\phi_{F,\,t}\,\psi_{PbBy,\,F} + N_{Nb,\,F,\,t}\,\phi_{F,\,t}\,\gamma_{t-1} + \\
& N_{By,\,F,\,t}\,\phi_{F,\,t}\,\phi_{By,F,\,t} + \\
& N_{Bc1,\,F,\,t}\,\phi_{F,\,t}\,\phi_{By,F,\,t} + \\
& N_{Bc2,\,F,\,t}\,\phi_{F,\,t}\,\phi_{By,F,\,t}\,F_t + \\
& N_{Bc2,\,F,\,t}\,\phi_{F,\,t}\,\phi_{By,F,\,t}\,(1-F_t) + N_{Bc3,\,F,\,t}\,\phi_{F,\,t} + N_{Bc1,\,F,\,t}\,\phi_{F,\,t}\,(1-\phi_{By,F,\,t}) + \\
& N_{By,\,F,\,t}\,\phi_{F,\,t}\,(1-\phi_{By,F,\,t}) + N_{Nb,\,F,\,t}\,\phi_{F,\,t}\,(1-\gamma_t) + \\
& N_{Bc2,\,F,\,t}\,\phi_{F,\,t}\,\phi_{By,F,\,t}\,(1-F_t)\,0.5 + N_{Bc3,\,F,\,t}\,\phi_{JuvSub,\,M,\,t}\,0.5 + N_{Juv,\,M,\,t}\,\phi_{JuvSub,\,M,\,t}\,(1-\psi_M) + \\
& N_{Juv,\,M,\,t}\,\phi_{JuvSub,\,M,\,t}\,\psi_M + N_{Sub,\,M,\,t}\,\phi_{JuvSub,\,M,\,t}\,(1-\psi_M) + \\
& N_{Sub,\,M,\,t}\,\phi_{JuvSub,\,M,\,t}\,\psi_M + N_{AdNt,\,M,\,t}\,\phi_{Ad,\,M,\,t}\,(1-\psi_M) + \\
& N_{AdNt,\,M,\,t}\,\phi_{Ad,\,M,\,t}\,\psi_M + N_{Ad,\,M,\,t}\,\phi_{Ad,\,M,\,t} + \\
& N_{Tot,\,FM,\,t}\,\iota_{FM,\,t}
\end{aligned} \tag{S75}$$

where the first row of eq. S75 is referred to juvenile females (excluding immigrants), the second row is referred to pre-breeding females, the third row to breeding females with a young-of-the-year, the fourth row to breeding females with a 1-year-old calf, the fifth row to breeding females with a 2-year-old calf, the sixth row to breeding females with a 3-year-old calf, the seventh and eighth row to non-breeding females, the ninth row to juvenile males (excluding immigrants), the tenth row to subadult males, the eleventh row to not toothed adult males, the twelfth row to toothed adult males, and the last row to immigrants of both sexes, with $\iota_{FM,\,t}$ denoting immigration rate for the whole population (eq. S72). Remember that $N_{Tot,\,FM,\,t} = N_{Juv,\,F,\,t} + N_{Pb,\,F,\,t} + N_{By,\,F,\,t} + N_{Bc1,\,F,\,t} + N_{Bc2,\,F,\,t} + N_{Bc3,\,F,\,t} + N_{Nb,\,F,\,t} + N_{Juv,\,M,\,t} + N_{Sub,\,M,\,t} + N_{AdNt,\,M,\,t} + N_{Ad,\,M,\,t}$.

The population vector in year $t$ is:

$$\boldsymbol{N_t} = \{N_{Juv,\,F,\,t},\, N_{Pb,\,F,\,t},\, N_{By,\,F,\,t},\, N_{Bc1,\,F,\,t},\, N_{Bc2,\,F,\,t},\, N_{Bc3,\,F,\,t},\, N_{Nb,\,F,\,t},\, N_{Juv,\,M,\,t},\, N_{Sub,\,M,\,t},\, N_{AdNt,\,M,\,t},\, N_{Ad,\,M,\,t}\}. \tag{S76}$$

## S.3 Selection of multievent models for the two sexes

For each sex, the multievent model (Pradel 2005) implemented in the integrated population model (IPM) was first selected using program E-SURGE (Choquet et al. 2009). A multievent model uses row stochastic matrices for initial states, transitions and events. Below we report details about model structure, implementation and selection for each sex.

### S.3.1 Goodness-of-fit (GOF)

There is no specific GOF test for multievent models. We thus assessed the fit of the general mark-recapture assumptions to our data by assessing the GOF of the single state Cormack-Jolly-Seber (CJS) model (Cormack 1964; Jolly 1965; Seber 1965) using program U-CARE (Choquet et al. 2005) and the R package R2ucare (Gimenez et al. 2017). The results of the GOF tests no detectable lack of fit of the CJS model in either dataset (females: $\chi^2$ = 21.30, df = 45, $P$ = 0.999; males: $\chi^2$ = 32.81, df = 47, $P$ = 0.942). In addition, there was no evidence for overdispersion in either dataset.

### S.3.2 Females

We assumed eight states for females: 'juvenile' ($Juv$), 'pre-breeder' ($Pb$), 'breeder with a young-of-the-year' ($By$), 'breeder with a 1-year-old calf' ($Bc1$), 'breeder with a 2-year-old calf' ($Bc2$), 'breeder with a 3-year-old calf' ($Bc3$), 'non-breeder' ($Nb$), and 'dead' ($D$). Seven types of mutually exclusive events could be observed and were arbitrarily coded as follows: '0' = 'not seen', '1' = 'seen as juvenile', '2' = 'seen as pre-breeder', '3' = 'seen as breeder with a young-of-the-year', '4' = 'seen as breeder with a 1-year-old or older calf', '5' = 'seen as non-breeder', '6' = 'seen alone'. The elementary matrices used in E-SURGE are as follows, with departure states in rows and intermediate/arriving states or events in columns. Parameter text notation used in the Gepat interface of program E-SURGE is reported.
Initial state probabilities:

| | Juv | Pb | By | Bc1 | Bc2 | Bc3 | Nb |
|---|---|---|---|---|---|---|---|
| | pi1 | pi2 | pi3 | pi4 | pi5 | pi6 | 1-(∑1:6 pi) |

Survival of individuals older than calves (here 'phi' corresponds to $\phi_F$ in the previous sections):

| | Juv | Pb | By | Bc1 | Bc2 | Bc3 | Nb | D |
|---|---|---|---|---|---|---|---|---|
| Juv | phi | 0 | 0 | 0 | 0 | 0 | 0 | 1-phi |
| Pb | 0 | phi | 0 | 0 | 0 | 0 | 0 | 1-phi |
| By | 0 | 0 | phi | 0 | 0 | 0 | 0 | 1-phi |
| Bc1 | 0 | 0 | 0 | phi | 0 | 0 | 0 | 1-phi |
| Bc2 | 0 | 0 | 0 | 0 | phi | 0 | 0 | 1-phi |
| Bc3 | 0 | 0 | 0 | 0 | 0 | phi | 0 | 1-phi |
| Nb | 0 | 0 | 0 | 0 | 0 | 0 | phi | 1-phi |
| D | 0 | 0 | 0 | 0 | 0 | 0 | 0 | 1 |

Transition probabilities (here 'psi' corresponds to $\psi_{JuvPB,\,F}$ and $\psi_{PbBy,\,F}$ in the previous sections, and By* denotes the intermediate state for individuals that recruit as breeders):

| | Juv | Pb | By* | By | Bc1 | Bc2 | Bc3 | Nb | D |
|---|---|---|---|---|---|---|---|---|---|
| Juv | 1-psi | psi | 0 | 0 | 0 | 0 | 0 | 0 | 0 |
| Pb | 0 | 1-psi | psi | 0 | 0 | 0 | 0 | 0 | 0 |
| By | 0 | 0 | 0 | 1 | 0 | 0 | 0 | 0 | 0 |
| Bc1 | 0 | 0 | 0 | 0 | 1 | 0 | 0 | 0 | 0 |
| Bc2 | 0 | 0 | 0 | 0 | 0 | 1 | 0 | 0 | 0 |
| Bc3 | 0 | 0 | 0 | 0 | 0 | 0 | 1 | 0 | 0 |
| Nb | 0 | 0 | 0 | 0 | 0 | 0 | 0 | 1 | 0 |
| D | 0 | 0 | 0 | 0 | 0 | 0 | 0 | 0 | 1 |

Survival of young-of-the-year and calves ($\phi_{Bc,F,\,t}$, here in the matrix is denoted by 'phi_c'; Nb* denotes the intermediate state for females that lose their young/calf between $t$ and $t+1$, becoming non-breeder at $t+1$):

| | Juv | Pb | By | Bc1 | Bc2 | Bc3 | Nb* | Nb | D |
|---|---|---|---|---|---|---|---|---|---|
| Juv | 1 | 0 | 0 | 0 | 0 | 0 | 0 | 0 | 0 |
| Pb | 0 | 1 | 0 | 0 | 0 | 0 | 0 | 0 | 0 |
| By* | 0 | 0 | 1 | 0 | 0 | 0 | 0 | 0 | 0 |
| By | 0 | 0 | 0 | phi_c | 0 | 0 | 1-phi_c | 0 | 0 |
| Bc1 | 0 | 0 | 0 | 0 | phi_c | 0 | 1-phi_c | 0 | 0 |
| Bc2 | 0 | 0 | 0 | 0 | 0 | phi_c | 1-phi_c | 0 | 0 |
| Bc3 | 0 | 0 | 0 | 0 | 0 | 0 | 1 | 0 | 0 |
| Nb | 0 | 0 | 0 | 0 | 0 | 0 | 0 | 1 | 0 |
| D | 0 | 0 | 0 | 0 | 0 | 0 | 0 | 0 | 1 |

Breeding (here 'gamma' corresponds to breeding probability $\gamma_t$.):

| | Juv | Pb | By | Bc1 | Bc2 | Bc3 | Nb | D |
|---|---|---|---|---|---|---|---|---|
| Juv | 1 | 0 | 0 | 0 | 0 | 0 | 0 | 0 |
| Pb | 0 | 1 | 0 | 0 | 0 | 0 | 0 | 0 |
| By | 0 | 0 | 1 | 0 | 0 | 0 | 0 | 0 |
| Bc1 | 0 | 0 | 0 | 1 | 0 | 0 | 0 | 0 |
| Bc2 | 0 | 0 | 0 | 0 | 1 | 0 | 0 | 0 |
| Bc3 | 0 | 0 | 0 | 0 | 0 | 1 | 0 | 0 |
| Nb* | 0 | 0 | 0 | 0 | 0 | 0 | 1 | 0 |
| Nb | 0 | 0 | gamma | 0 | 0 | 0 | 1-gamma | 0 |
| D | 0 | 0 | 0 | 0 | 0 | 0 | 0 | 1 |

Encounter (here 'p' corresponds to encounter/resighting probability $p_{F,t}$, conditional on the true state of the individual):

| | not detected | detect Juv | detect Pb | detect By | detect Bc1 | detect Bc2 | detect Bc3 | detect Nb |
|---|---|---|---|---|---|---|---|---|
| Juv | 1-p | p | 0 | 0 | 0 | 0 | 0 | 0 |
| Pb | 1-p | 0 | p | 0 | 0 | 0 | 0 | 0 |
| By | 1-p | 0 | 0 | p | 0 | 0 | 0 | 0 |
| Bc1 | 1-p | 0 | 0 | 0 | p | 0 | 0 | 0 |
| Bc2 | 1-p | 0 | 0 | 0 | 0 | p | 0 | 0 |
| Bc3 | 1-p | 0 | 0 | 0 | 0 | 0 | p | 0 |
| Nb | 1-p | 0 | 0 | 0 | 0 | 0 | 0 | p |
| D | 1 | 0 | 0 | 0 | 0 | 0 | 0 | 0 |

Observation (here 'delta' and 'epsilon' correspond to the probability that the state of the individual was ascertained during observation, for pre-breeding and non-breeding females, $\delta_F$):

| | 0 | 1 | 2 | 3 | 4 | 5 | 6 |
|---|---|---|---|---|---|---|---|
| | not seen | seen Juv | seen Pb | seen By | seen Bc1+ | seen NB | seen uncertain |
| not detected | 1 | 0 | 0 | 0 | 0 | 0 | 0 |
| detect Juv | 0 | 1 | 0 | 0 | 0 | 0 | 0 |
| detect Pb | 0 | 0 | delta | 0 | 0 | 0 | 1-delta |
| detect By | 0 | 0 | 0 | 1 | 0 | 0 | 0 |
| detect Bc1 | 0 | 0 | 0 | 0 | 1 | 0 | 0 |
| detect Bc2 | 0 | 0 | 0 | 0 | 1 | 0 | 0 |
| detect Bc3 | 0 | 0 | 0 | 0 | 1 | 0 | 0 |
| detect Nb | 0 | 0 | 0 | 0 | 0 | epsilon | 1-epsilon |

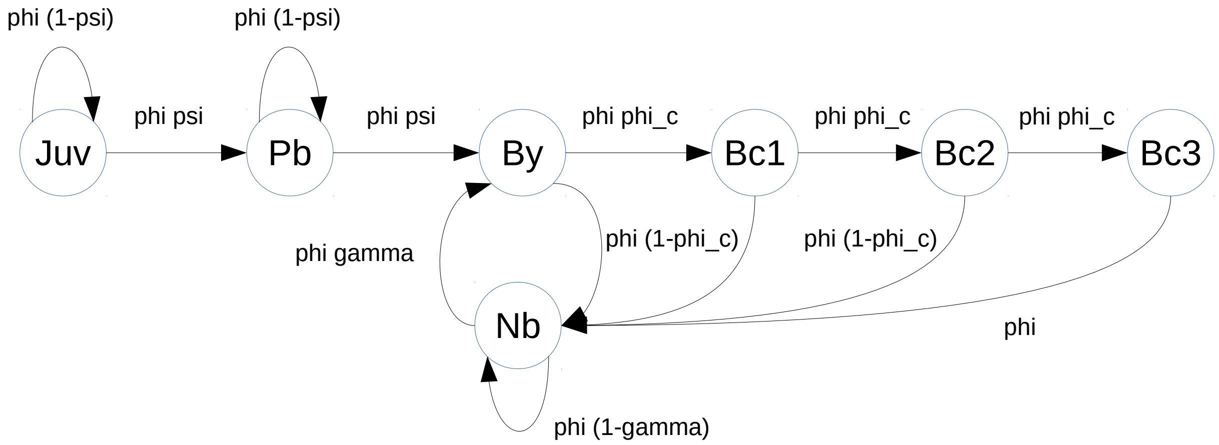


Figure S4 General pattern of transitions between states for females.

### S.3.2.1 *Model selection*

Model selection was based on the Akaike Information Criterion (AIC) given there was no evidence for overdispersion in either data set. In the case of models with a difference in AIC <2 units from the lowest AIC score, we selected the most parsimonious model (i.e., the one with the lower number of identifiable parameters). For females, we used a 2-stage model selection process (Grosbois and Tavecchia 2003). First, we modelled detection and observation probabilities while keeping survival probabilities fully parameterized. Next, we used the best structure for detection and observation probabilities identified in the first stage to model survival probabilities. A total of 18 models testing for survival/encounter differences among age and stage classes and the effect of field effort on resighting probabilities were compared across the two stages of the model selection procedure (Table S8-S9).

Table S8: Stage 1: Selection of the detection and observation process. Definition of model constraints using the GEMACO tool, which is part of E-SURGE, is given.

| | | **Model** | | | | | | |
|---|---|---|---|---|---|---|---|---|
| | **GEMACO** | 1 | 2 | 3 | 4 | 5 | 6 | 7 |
| **Initial state probabilities** | | | | | | | | |
| Constant | intercept | x | | | | | | |
| **Survival of individuals older than calves** | | | | | | | | |
| Constant | intercept | | | | | | | |
| Juv vs others (PB, B*, NB) | from(1,2:7) | | | | | | | |
| Juv vs PB vs others (B*, NB) | from(1,2,3:7) | | | | | | | |
| Juv, PB vs others (B*, NB) | from(1:2,3:7) | | | | | | | |
| Juv, PB vs B* vs NB | from(1:2,3:6,7) | | | | | | | |
| Juv vs PB vs B* vs NB | from(1,2,3:6,7) | x | | | | | | |
| **Transitions** | | | | | | | | |
| JUV to PB vs PB to By | from(1,2) | x | | | | | | |
| **Survival of young-of-the-year and calves** | | | | | | | | |
| By vs Bc1,Bc2 | from(4,5:6) | x | | | | | | |
| **Breeding (already recruited individuals)** | | | | | | | | |
| Constant | intercept | x | | | | | | |
| **Encounter** | | | | | | | | |
| Constant | firste+nexte.[i] | x | | | | | | |
| effort | firste+nexte.[i+t*x] | | x | | | | | |
| Juv vs others (PB, B*, NB) | firste+nexte.[from(1,2:7)+t*x] | | | x | | | | |
| Juv vs PB vs others (B*, NB) | firste+nexte.[from(1,2,3:7)+t*x] | | | | x | | | |
| Juv, PB vs others (B*, NB) | firste+nexte.[from(1:2,3:7)+t*x] | | | | | x | | |
| Juv, PB vs B* vs NB | firste+nexte.[from(1:2,3:6,7)+t*x] | | | | | | x | |
| Juv vs PB vs B* vs NB | firste+nexte.[from(1,2,3:6,7)+t*x] | | | | | | | x |
| **Observation** | | | | | | | | |
| Constant | intercept | x | | | | | | |

Table S9: Stage 2: Selection of the state process (survival)

| | | Model | | | | | | | | | | | |
|---|---|---|---|---|---|---|---|---|---|---|---|---|---|
| | **GEMACO** | 8 | 9 | 10 | 11 | 12 | 13 | 14 | 15 | 16 | 17 | 18 | 2 |
| **Initial state probabilities** | | | | | | | | | | | | | |
| Constant | intercept | x | | | | | | | | | | | |
| **Survival of individuals older than calves** | | | | | | | | | | | | | |
| Constant | intercept | x | | | | | | x | | | | | |
| Juv vs others (PB, B*, NB) | from(1,2:7) | | x | | | | | | x | | | | |
| Juv vs PB vs others (B*, NB) | from(1,2,3:7) | | | x | | | | | | x | | | |
| Juv, PB vs others (B*, NB) | from(1:2,3:7) | | | | x | | | | | | x | | |
| Juv, PB vs B* vs NB | from(1:2,3:6,7) | | | | | x | | | | | | x | |
| Juv vs PB vs B* vs NB | from(1,2,3:6,7) | | | | | | x | | | | | | x |
| **Transitions** | | | | | | | | | | | | | |
| JUV to PB vs PB to By | from(1,2) | x | | | | | | | | | | | |
| **Survival of young-of-the-year and calves** | | | | | | | | | | | | | |
| Constant | intercept | x | x | x | x | x | x | | | | | | |
| By vs Bc1,Bc2 | from(4,5:6) | | | | | | | x | x | x | x | x | x |
| **Breeding (already recruited individuals)** | | | | | | | | | | | | | |
| Constant | intercept | x | | | | | | | | | | | |
| **Encounter** | | | | | | | | | | | | | |
| effort | firste+nexte.[i+t*x] | x | | | | | | | | | | | |
| **Observation** | | | | | | | | | | | | | |
| Constant | intercept | x | | | | | | | | | | | |

Table S10: Summary results of the multievent mark-recapture analysis in females. Notation: np, number of estimable parameters; dev, deviance; AICc, Akaike information criterion corrected by sample size; ΔAICc, the AICc difference between the current model and the one with the lowest AICc value. The structure of Model 14 was implemented in the integrated population model.

| Model | np | dev | AICc | ΔAICc |
|---|---|---|---|---|
| Model 14 | 10 | 787.695 | 807.695 | 0.000 |
| Model 17 | 11 | 786.639 | 808.639 | 0.944 |
| Model 15 | 11 | 787.151 | 809.151 | 1.456 |
| Model 18 | 12 | 785.795 | 809.795 | 2.100 |
| Model 16 | 12 | 786.639 | 810.639 | 2.944 |
| Model 2 | 13 | 785.795 | 811.795 | 4.100 |
| Model 5 | 14 | 784.078 | 812.078 | 4.383 |
| Model 6 | 15 | 783.350 | 813.350 | 5.655 |
| Model 4 | 15 | 783.639 | 813.639 | 5.944 |
| Model 3 | 14 | 785.661 | 813.661 | 5.966 |
| Model 7 | 16 | 782.880 | 814.880 | 7.185 |
| Model 8 | 9 | 800.011 | 818.011 | 10.316 |
| Model 11 | 10 | 798.966 | 818.966 | 11.271 |
| Model 9 | 10 | 799.466 | 819.466 | 11.771 |
| Model 12 | 11 | 798.098 | 820.098 | 12.403 |
| Model 10 | 11 | 798.966 | 820.966 | 13.271 |
| Model 13 | 12 | 798.098 | 822.098 | 14.403 |
| Model 1 | 12 | 802.539 | 826.539 | 18.844 |

### S.3.3 Males

We assumed five states for males: 'juvenile' ($Juv$), 'subadult' ($Sub$), 'not toothed adult male' ($AdNt$), 'toothed adult male' ($Ad$), and 'dead' ($D$). Six types of mutually exclusive events could be observed and were arbitrarily coded as follows: '0' = 'not seen', '1' = 'seen as juvenile', '2' = 'seen as subadult', '3' = 'seen as not toothed adult', '4' = 'seen as toothed adult', '5' = 'seen as adult'.

The elementary matrices used in E-SURGE are as follows, with departure states in rows and intermediate/arriving states or events in columns.

Initial state probabilities:

| | Juv | Sub | AdNt | Ad |
|---|---|---|---|---|
| | pi1 | pi2 | pi3 | 1-(∑1:3 pi) |

Survival; here 'phi' corresponds to the apparent survival probability for juvenile and subadult males ($\phi_{JuvSub,\,M,\,t}$) or to the apparent survival probability for adult males (both not toothed and toothed; $\phi_{Ad,\,M,\,t}$) of the multievent model implemented in the IPM:

| | Juv | Sub | AdNt | Ad | D |
|---|---|---|---|---|---|
| Juv | phi | 0 | 0 | 0 | 1-phi |
| Sub | 0 | phi | 0 | 0 | 1-phi |
| AdNt | 0 | 0 | phi | 0 | 1-phi |
| Ad | 0 | 0 | 0 | phi | 1-phi |
| D | 0 | 0 | 0 | 0 | 1 |

Transitions (here 'psi' corresponds to transition probability $\psi_M$):

| | Juv | Sub | AdNt | Ad | D |
|---|---|---|---|---|---|
| Juv | 1-psi | psi | 0 | 0 | 0 |
| Sub | 0 | 1-psi | psi | 0 | 0 |
| AdNt | 0 | 0 | 1-psi | psi | 0 |
| Ad | 0 | 0 | 0 | 1 | 0 |
| D | 0 | 0 | 0 | 0 | 1 |

Encounter; here ‘p’ corresponds to male encounter (resighting) probability for juveniles ($p_{Juv,\,M,\,t}$), subadults ($p_{Sub,\,M,\,t}$), not toothed adults ($p_{AdNt,\,M,\,t}$), toothed adults ($p_{Ad,\,M,\,t}$) of the multievent model implemented in the IPM:

| | not detected | detect Juv | detect Sub | detect AdNt | detect Ad |
|---|---|---|---|---|---|
| Juv | 1-p | p | 0 | 0 | 0 |
| Sub | 1-p | 0 | p | 0 | 0 |
| AdNt | 1-p | 0 | 0 | p | 0 |
| Ad | 1-p | 0 | 0 | 0 | p |
| D | 1 | 0 | 0 | 0 | 0 |

Observation; here ‘delta’ corresponds to the probabilities that the state of the individual was ascertained during observation, for not toothed and toothed adult males ($\delta_{AdNt,\,M}$ and $\delta_{Ad,\,M}$, respectively) reported in the multievent model implemented in the IPM:

| | 0 | 1 | 2 | 3 | 4 | 5 |
|---|---|---|---|---|---|---|
| | not seen | seen Juv | seen Sub | seen AdNt | seen Ad | seen Ad not ascertained |
| not detected | 1 | 0 | 0 | 0 | 0 | 0 |
| detect Juv | 0 | 1 | 0 | 0 | 0 | 0 |
| detect Sub | 0 | 0 | 1 | 0 | 0 | 0 |
| detect AdNt | 0 | 0 | 0 | delta | 0 | 1-delta |
| detect Ad | 0 | 0 | 0 | 0 | delta | 1-delta |

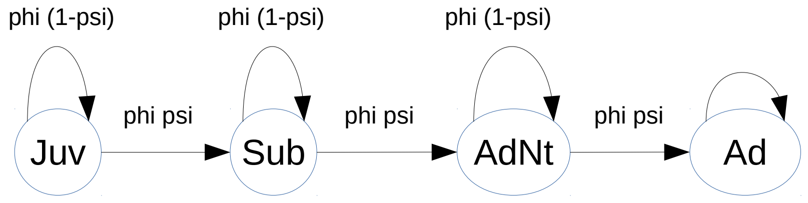


Figure S5 General pattern of transitions between states for males.

#### S.3.3.1 *Model selection*

Model selection was based on the Akaike's Information Criterion (AIC) given there was no evidence for overdispersion in either dataset. In the case of models with a difference in AIC <2 units from the lowest AIC score, we selected the most parsimonious model (i.e., the one with the lower number of identifiable parameters). For males, we used a 3-stage model selection process. First, we modelled detection and observation probabilities while keeping survival and transitions probabilities fully parameterized. Next, we used the best structure for detection and observation probabilities identified in the first stage to model survival probabilities. Finally, we modelled transition probabilities while keeping all other parameters parameterized according to the best models identified. A total of 15 models were compared across the three stages of the model selection procedure (Table S11, S12, S13).

Table S11: Stage 1: Selection of the detection and observation process. Definition of model constraints using the GEMACO tool, which is part of E-SURGE, is given.

| | | **Model** | | | | | | | | | | | |
|---|---|---|---|---|---|---|---|---|---|---|---|---|---|
| | **GEMACO** | 1 | 2 | 3 | 4 | 5 | 6 | 7 | 8 | 9 | 10 | 11 | 12 |
| **Initial state probabilities** | | | | | | | | | | | | | |
| Constant | intercept | x | | | | | | | | | | | |
| **Survival** | | | | | | | | | | | | | |
| Constant | intercept | | | | | | | | | | | | |
| Juv, Sub vs AdNt, Ad | from(1:2,3:4) | | | | | | | | | | | | |
| Juv vs Sub vs AdNt, Ad | from(1,2,3:4) | | | | | | | | | | | | |
| Juv vs Sub vs AdNt vs Ad | from(1,2,3,4) | x | | | | | | | | | | | |
| **Transitions** | | | | | | | | | | | | | |
| Constant | intercept | | | | | | | | | | | | |
| Juv to Sub vs Sub to AdNt vs AdNt to Ad | from(1,2,3) | x | | | | | | | | | | | |
| **Encounter** | | | | | | | | | | | | | |
| Constant | firste+nexte.[i] | x | x | | | | | | | | | | |
| effort | firste+nexte.[i+t*x] | | | x | x | | | | | | | | |
| Juv, Sub vs AdNt, Ad + effort | firste+nexte.[from(1:2,3:4)+t*x] | | | | | x | x | | | | | | |
| Juv vs Sub vs AdNt, Ad + effort | firste+nexte.[from(1,2,3:4)+t*x] | | | | | | | x | x | | | | |
| Juv, Sub vs AdNt vs Ad + effort | firste+nexte.[from(1:2,3,4)+t*x] | | | | | | | | | x | x | | |
| Juv vs Sub vs AdNt vs Ad + effort | firste+nexte.[from(1,2,3,4)+t*x] | | | | | | | | | | | x | x |
| **Observation** | | | | | | | | | | | | | |
| Constant | intercept | x | | x | | x | | x | | x | | x | |
| detect AdNt vs detect Ad | from | | x | | x | | x | | x | | x | | x |

Table S12: Stage 2: Selection of the state process (survival).

| | | Model | | | |
|---|---|---|---|---|---|
| | **GEMACO** | 13 | 14 | 15 | 12 |
| **Initial state probabilities** | | | | | |
| Constant | intercept | x | | | |
| **Survival** | | | | | |
| Constant | intercept | x | | | |
| Juv, Sub vs AdNt, Ad | from(1:2,3:4) | | x | | |
| Juv vs Sub vs AdNt, Ad | from(1,2,3:4) | | | x | |
| Juv vs Sub vs AdNt vs Ad | from(1,2,3,4) | | | | x |
| **Transitions** | | | | | |
| Constant | intercept | | | | |
| Juv to Sub vs Sub to AdNt vs AdNt to Ad | from(1,2,3) | x | | | |
| **Encounter** | | | | | |
| Juv vs Sub vs AdNt vs Ad + effort | firste+nexte.[from(1,2,3,4)+t*x] | x | | | |
| **Observation** | | | | | |
| detect AdNt vs detect Ad | from | x | | | |

Table S13: Stage 3: Selection of the state process (transitions).

| | | Model | |
|---|---|---|---|
| | **GEMACO** | 15 | 14 |
| **Initial state probabilities** | | | |
| Constant | intercept | x | |
| **Survival** | | | |
| Constant | intercept | | |
| Juv, Sub vs AdNt, Ad | from(1:2,3:4) | x | |
| Juv vs Sub vs AdNt, Ad | from(1,2,3:4) | | |
| Juv vs Sub vs AdNt vs Ad | from(1,2,3,4) | | |
| **Transitions** | | | |
| Constant | intercept | x | |
| Juv to Sub vs Sub to AdNt vs AdNt to Ad | from(1,2,3) | | x |
| **Encounter** | | | |
| Juv vs Sub vs AdNt vs Ad + effort | firste+nexte.[from(1,2,3,4)+t*x] | x | |
| **Observation** | | | |
| detect AdNt vs detect Ad | from | x | |

Table S14: Summary results of the multievent mark-recapture analysis in males. Notation: np, number of estimable parameters; dev, deviance; AICc, Akaike information criterion corrected by sample size; ΔAICc, the AICc difference between the current model and the one with the lowest AICc value. The structure of Model 14 was implemented in the integrated population model.

| Model | np | dev | AICc | ΔAICc |
|---|---|---|---|---|
| Model15 | 11 | 1384.9652 | 1406.9652 | 0 |
| Model14 | 14 | 1383.7329 | 1411.7329 | 4.7677 |
| Model12 | 15 | 1383.6991 | 1413.6991 | 6.7339 |
| Model13 | 12 | 1390.6404 | 1414.6404 | 7.6752 |
| Model10 | 14 | 1388.3789 | 1416.3789 | 9.4137 |
| Model8 | 14 | 1389.2118 | 1417.2118 | 10.2466 |
| Model6 | 13 | 1396.4762 | 1422.4762 | 15.511 |
| Model4 | 12 | 1406.4947 | 1430.4947 | 23.5295 |
| Model11 | 14 | 1407.198 | 1435.198 | 28.2328 |
| Model9 | 13 | 1411.5015 | 1437.5015 | 30.5363 |
| Model7 | 13 | 1413.1241 | 1439.1241 | 32.1589 |
| Model5 | 12 | 1420.5712 | 1444.5712 | 37.606 |
| Model3 | 11 | 1430.7078 | 1452.7078 | 45.7426 |
| Model2 | 11 | 1471.8569 | 1493.8569 | 86.8917 |
| Model1 | 10 | 1496.0964 | 1516.0964 | 109.1312 |

## S.4 JAGS model code for the integrated population model

Links to model equations are reported in the model script.

```
model{
##############################################
# Population count data
##############################################
############################
# System process
############################
### Initial population sizes
#---------------------------------------
#---- FEMALES
#---------------------------------------
#number of juveniles from 2yr weaned
N_juv_tot_part1a[1] ~ dcat(p_n_juv_tot_part1a_inits[])
N_juv_f_part1a[1] <- round(N_juv_tot_part1a[1]*0.5)
#total number of weaned 3yr calves
N_w3[1] = N_bc3_f[1]
#number of females weaned at 3 years
N_w3_f[1] <- round(N_w3[1]*0.5)
#number of dead calves between 2nd and 3rd year of life (mother alive)
Ndead23[1] ~ dcat(p_n_dead23_inits[])
#number of dead mothers when calf is between 2nd and 3rd year of life
Ndead_f[1] <- 0
#number of juveniles from 3yr weaned
N_juv_f_part1b[1] ~ dcat(p_n_juv_f_part1b_inits[])
#number of juveniles from juveniels of previous year
N_juv_f_part2[1] ~ dcat(p_n_juv_f_part2_inits[])
#number of juvenile females (immigrants excluded)
N_juv_f_NoImm[1] <- N_juv_f_part1a[1] + N_juv_f_part1b[1] + N_juv_f_part2[1]
#number of juvenile females
N_juv_f[1] <- N_juv_f_part1a[1] + N_juv_f_part1b[1] + N_juv_f_part2[1] + N_imm_f[1]
#number of pre-breeders
N_pb_f_part1[1] ~ dcat(p_n_pb_f_part1_inits[])
N_pb_f_part2[1] ~ dcat(p_n_pb_f_part2_inits[])
N_pb_f[1] <- N_pb_f_part1[1] + N_pb_f_part2[1]
#number of breeders with young of the year
N_by_f_part1[1] ~ dcat(p_n_by_f_part1_inits[])
```

```
N_by_f_part2[1] ~ dcat(p_n_by_f_part2_inits[])
N_by_f[1] <- N_by_f_part1[1] + N_by_f_part2[1]
#number of breeders with a 1yr calf
N_bc1_f[1] ~ dcat(p_n_bc1_f_inits[])
#number of breeders with a 2yr calf
N_bc2_f[1] ~ dcat(p_n_bc2_f_inits[])
#number of breeders with a 3yr calf
N_bc3_f[1] ~ dcat(p_n_bc3_f_inits[])
#number of non-breeders
N_nb_f_part1a[1] = N_juv_tot_part1a[1]
N_nb_f_part1b[1] ~ dcat(p_n_nb_f_part1b_inits[])
N_nb_f_part1c[1] = Ndead23[1]
N_nb_f_part1d[1] ~ dcat(p_n_nb_f_part1d_inits[])
N_nb_f_part1e[1] ~ dcat(p_n_nb_f_part1e_inits[])
N_nb_f_part2[1] ~ dcat(p_n_nb_f_part2_inits[])
N_nb_f[1] <- N_nb_f_part1a[1] + N_nb_f_part1b[1] +
             N_nb_f_part1c[1] + N_nb_f_part1d[1] +
             N_nb_f_part1e[1] + N_nb_f_part2[1]
# number of immigrants
N_imm_f[1] ~ dbin(0.5, N_Imm[1])
#----------------------------------------
#---- MALES
#----------------------------------------
#number of juveniles from individuals weaned between 2nd and 3rd year of life (even sex ratio)
N_juv_m_part1a[1] = N_juv_tot_part1a[1] - N_juv_f_part1a[1]
#number of males weaned at 3 years
N_w3_m[1] <- N_w3[1] - N_w3_f[1]
#number of juveniles from 3yr weaned
N_juv_m_part1b[1] ~ dcat(p_n_juv_m_part1b_inits[])
#number of juveniles from juveniels of previous year
N_juv_m_part2[1] ~ dcat(p_n_juv_m_part2_inits[])
#number of juvenile males (immigrants excluded)
N_juv_m_NoImm[1] <- N_juv_m_part1a[1] + N_juv_m_part1b[1] + N_juv_m_part2[1]
#number of juvenile males
N_juv_m[1] <- N_juv_m_part1a[1] + N_juv_m_part1b[1] + N_juv_m_part2[1] + N_imm_m[1]
# number of subadults
N_sub_m_part1[1] ~ dcat(p_n_sub_m_part1_inits[])
N_sub_m_part2[1] ~ dcat(p_n_sub_m_part2_inits[])
N_sub_m[1] <- N_sub_m_part1[1] + N_sub_m_part2[1]
# number of adults not toothed
```

```
N_adnt_m_part1[1] ~ dcat(p_n_adnt_m_part1_inits[])
N_adnt_m_part2[1] ~ dcat(p_n_adnt_m_part2_inits[])
N_adnt_m[1] <- N_adnt_m_part1[1] + N_adnt_m_part2[1]
# number of toothed adults
N_ad_m_part1[1] ~ dcat(p_n_ad_m_part1_inits[])
N_ad_m_part2[1] ~ dcat(p_n_ad_m_part2_inits[])
N_ad_m[1] <- N_ad_m_part1[1] + N_ad_m_part2[1]
# number of immigrants
N_imm_m[1] <- N_Imm[1] - N_imm_f[1]

#-----------------------------------------
#---- TOTALS
#-----------------------------------------
#total number of juveniles (immigrants included)
N_juv[1] <- N_juv_f[1] + N_juv_m[1]

#total number of non-reproductive individuals (older than juveniles)
N_nr[1] <- N_pb_f[1] + N_nb_f[1] +
               N_sub_m[1] + N_adnt_m[1]

#total number of reproductive females (equal to)
#total number of young and calves
N_br[1] <- N_by_f[1] + N_bc1_f[1] + N_bc2_f[1] + N_bc3_f[1]

#total number of females and males
Ntot_f[1] <- N_juv_f[1] + N_pb_f[1] + N_br[1] + N_nb_f[1]
Ntot_m[1] <- N_juv_m[1] + N_sub_m[1] + N_adnt_m[1] + N_ad_m[1]

#total number of individuals
Ntot[1] <- N_juv[1] + N_nr[1] + N_br[1]

#total number of immigrants
N_Imm[1] ~ dcat(p_n_imm_inits[])

for(t in 2:nyears){
        #-----------------------------------------
        #---- FEMALES and youngs with mother
        #-----------------------------------------
        #Calf fidelity (i.e. not weaning)
        fid[t-1] <- phi_Bc[t-1] / phi_By[t-1]
```

```
#probability of weaning between 2nd and 3rd year of life
pi1[t-1] <- phi[t-1]*phi_By[t-1]*(1-fid[t-1]) #eq. S2
#number of juveniles from individuals weaned between 2nd and 3rd year of life
N_juv_tot_part1a[t] ~ dbin(pi1[t-1], N_bc2_f[t-1]) #eq. S1
#number of juvenile females from individuals weaned between 2nd and 3rd year
#of life (even sex ratio)
N_juv_f_part1a[t] ~ dbin(0.5, N_juv_tot_part1a[t]) #eq. S3

#probability of staying with mother and weaning at 3 years of life
pi2[t-1] <- (phi[t-1]*phi_By[t-1]*fid[t-1])/
            ((phi[t-1]*phi_By[t-1]*fid[t-1])+
             (phi[t-1]*(1-phi_By[t-1]))+(1-phi[t-1])) #eq. S5
Npi2[t-1] <- N_bc2_f[t-1]-N_juv_tot_part1a[t] #part of eq. S1
#number of breeders with a 3yr calf
N_bc3_f[t] ~ dbin(pi2[t-1], Npi2[t-1]) #eq. S4

#probability of dying for a calf with mother between 2nd and 3rd year of life
pi3[t-1] <- (phi[t-1]*(1-phi_By[t-1]))/
            ((phi[t-1]*(1-phi_By[t-1]))+(1-phi[t-1])) #eq. S7
Npi3[t-1] <- N_bc2_f[t-1]-N_juv_tot_part1a[t]-N_bc3_f[t] #part of eq. S6
Ndead23[t] ~ dbin(pi3[t-1], Npi3[t-1]) #eq. S6

#mothers died when calf is between 2nd and 3rd year of life
Npi4[t-1] <- N_bc2_f[t-1]-N_juv_tot_part1a[t]-N_bc3_f[t]-Ndead23[t] #part of eq. S8
Ndead_f[t] ~ dbin(1, Npi4[t-1]) #eq. S8

#total number of weaned 3yr calves
N_w3[t] = N_bc3_f[t] #eq. S9
#number of females weaned at 3 years of life
N_w3_f[t] ~ dbin(0.5, N_w3[t]) #eq. S10
#number of juveniles from 3yr weaned (even sex ratio)
N_juv_f_part1b[t] ~ dbin(phi[t-1], N_w3_f[t-1]) #eq. S11
#number of juveniles from juveniels of previous year
prob_juv_f_part2[t-1] <- phi[t-1] * (1-psi_JuPB) #part of eq. S12
N_juv_f_part2[t] ~ dbin(prob_juv_f_part2[t-1], N_juv_f[t-1]) #eq. S12
#number of juvenile females (immigrants excluded)
N_juv_f_NoImm[t] <- N_juv_f_part1a[t] + N_juv_f_part1b[t] + N_juv_f_part2[t]
#number of juvenile females
N_juv_f[t] <- N_juv_f_part1a[t] + N_juv_f_part1b[t] +
```

```
            N_juv_f_part2[t] + N_imm_f[t] #eq. S14
#number of pre-breeders
prob_pb_f_part1[t-1] <- phi[t-1] * psi_JuPB #part of eq. S15
N_pb_f_part1[t] ~ dbin(prob_pb_f_part1[t-1], N_juv_f[t-1]) #eq. S15
prob_pb_f_part2[t-1] <- phi[t-1] * (1-psi_PBBy) #part of eq. S16
N_pb_f_part2[t] ~ dbin(prob_pb_f_part2[t-1], N_pb_f[t-1]) #eq. S16
N_pb_f[t] <- N_pb_f_part1[t] + N_pb_f_part2[t] #eq. S17
#number of breeders with young of the year
prob_by_f_part1[t-1] <- phi[t-1] * psi_PBBy #part of eq. S18
N_by_f_part1[t] ~ dbin(prob_by_f_part1[t-1], N_pb_f[t-1]) #eq. S18
prob_by_f_part2[t-1] <- phi[t-1] * gamma[t-1] #part of eq. S19
N_by_f_part2[t] ~ dbin(prob_by_f_part2[t-1], N_nb_f[t-1]) #eq. S19
N_by_f[t] <- N_by_f_part1[t] + N_by_f_part2[t] #eq. S20
#number of breeders with a 1yr calf
prob_bc1_f[t-1] <- phi[t-1] * (phi_By[t-1]) #part of eq. S21
N_bc1_f[t] ~ dbin(prob_bc1_f[t-1], N_by_f[t-1]) #eq. S21
#number of breeders with a 2yr calf
prob_bc2_f[t-1] <- phi[t-1] * (phi_By[t-1]) #part of eq. S22
N_bc2_f[t] ~ dbin(prob_bc2_f[t-1], N_bc1_f[t-1]) #eq. S22

#number of non-breeders
N_nb_f_part1a[t] = N_juv_tot_part1a[t] #eq. S23
N_nb_f_part1b[t] ~ dbin(phi[t-1], N_bc3_f[t-1]) #eq. S24
#dead calf but mother alive
N_nb_f_part1c[t] = Ndead23[t] #eq. S25
prob_nb_f_part1d[t-1] <- phi[t-1] * (1-phi_By[t-1]) #part of eq. S26
N_nb_f_part1d[t] ~ dbin(prob_nb_f_part1d[t-1], N_bc1_f[t-1]) #eq. S26
prob_nb_f_part1e[t-1] <- phi[t-1] * (1-phi_By[t-1]) #part of eq. S27
N_nb_f_part1e[t] ~ dbin(prob_nb_f_part1e[t-1], N_by_f[t-1]) #eq. S27
prob_nb_f_part2[t-1] <- phi[t-1] * (1-gamma[t-1]) #part of eq. S28
N_nb_f_part2[t] ~ dbin(prob_nb_f_part2[t-1], N_nb_f[t-1]) #eq. S28
N_nb_f[t] <- N_nb_f_part1a[t] + N_nb_f_part1b[t] + N_nb_f_part1c[t] +
             N_nb_f_part1d[t] + N_nb_f_part1e[t] + N_nb_f_part2[t] #eq. S29
# number of immigrants
N_imm_f[t] ~ dbin(0.5, N_Imm[t]) #eq. S13

#--------------------------------------
#---- MALES
#--------------------------------------
#number of juveniles from individuals weaned between 2nd and 3rd year
```

```
#of life (even sex ratio)
N_juv_m_part1a[t] = N_juv_tot_part1a[t] - N_juv_f_part1a[t] #eq. S31
#number of males weaned at 3 years
N_w3_m[t] <- N_w3[t] - N_w3_f[t] #eq. S32
#number of juveniles from 3yr weaned
N_juv_m_part1b[t] ~ dbin(phi_JS_m[t-1], N_w3_m[t-1]) #eq. S33
#number of juveniles from juveniels of previous year
prob_juv_m_part2[t-1] <- phi_JS_m[t-1] * (1-psi_m)  #part of eq. S34
N_juv_m_part2[t] ~ dbin(prob_juv_m_part2[t-1], N_juv_m[t-1])  #eq. S34
#number of juvenile males (immigrants excluded)
N_juv_m_NoImm[t] <- N_juv_m_part1a[t] + N_juv_m_part1b[t] + N_juv_m_part2[t]
#number of juvenile males
N_juv_m[t] <- N_juv_m_part1a[t] + N_juv_m_part1b[t] +
              N_juv_m_part2[t] + N_imm_m[t]  #eq. S36
# number of subadults
prob_sub_m_part1[t-1] <- phi_JS_m[t-1] * psi_m #part of eq. S37
N_sub_m_part1[t] ~ dbin(prob_sub_m_part1[t-1], N_juv_m[t-1]) #eq. S37
prob_sub_m_part2[t-1] <- phi_JS_m[t-1] * (1-psi_m) #part of eq. S38
N_sub_m_part2[t] ~ dbin(prob_sub_m_part2[t-1], N_sub_m[t-1]) #eq. S38
N_sub_m[t] <- N_sub_m_part1[t] + N_sub_m_part2[t]  #eq. S39
# number of adults not toothed
prob_adnt_m_part1[t-1] <- phi_JS_m[t-1] * psi_m #part of eq. S40
N_adnt_m_part1[t] ~ dbin(prob_adnt_m_part1[t-1], N_sub_m[t-1]) #eq. S40
prob_adnt_m_part2[t-1] <- phi_Ad_m[t-1] * (1-psi_m) #part of eq. S41
N_adnt_m_part2[t] ~ dbin(prob_adnt_m_part2[t-1], N_adnt_m[t-1]) #eq. S41
N_adnt_m[t] <- N_adnt_m_part1[t] + N_adnt_m_part2[t] #eq. S42
# number of toothed adults
prob_ad_m_part1[t-1] <- phi_Ad_m[t-1] * psi_m #part of eq. S43
N_ad_m_part1[t] ~ dbin(prob_ad_m_part1[t-1], N_adnt_m[t-1]) #eq. S43
prob_ad_m_part2[t-1] <- phi_Ad_m[t-1] #part of eq. S44
N_ad_m_part2[t] ~ dbin(prob_ad_m_part2[t-1], N_ad_m[t-1]) #eq. S44
N_ad_m[t] <- N_ad_m_part1[t] + N_ad_m_part2[t] #eq. S45
# number of immigrants
N_imm_m[t] <- N_Imm[t] - N_imm_f[t]  #eq. S35

#----------------------------------------
#---- TOTALS
#----------------------------------------
#total number of juveniles
N_juv[t] <- N_juv_f[t] + N_juv_m[t] #eq. S48
```

```
        #total number of non-reproductive individuals (older than juveniles)
        N_nr[t] <- N_pb_f[t] + N_nb_f[t] +
                       N_sub_m[t] + N_adnt_m[t]  #eq.  S49

        #total number of reproductive females (equal to)
        #total number of young and calves
        N_br[t] <- N_by_f[t] + N_bc1_f[t] + N_bc2_f[t] + N_bc3_f[t]  #eq.  S50

        #total number of females and males
        Ntot_f[t] <- N_juv_f[t] + N_pb_f[t] + N_br[t] + N_nb_f[t]  #eq.  S51
        Ntot_m[t] <- N_juv_m[t] + N_sub_m[t] + N_adnt_m[t] + N_ad_m[t]  #eq.  S52

        #total number of individuals
        Ntot[t] <- N_juv[t] + N_nr[t] + N_br[t] #eq.  S53

        #total number of immigrants
        N_Imm[t] ~ dpois(omega_imm[t-1]) #eq.  S46
}#end state process


#----------------------------------------
#---- Immigration
#----------------------------------------
for(t in 1:(nyears-1)){
        # immigration
        log(omega_imm[t]) <- mu.omega_imm + eps_imm[t] #eq.  S47
        eps_imm[t] ~ dnorm(0, tau_imm)
}
mu.omega_imm <- log(mean.omega_imm)
mean.omega_imm ~ dunif(0.01,100)
tau_imm <- 1 / (sigma_imm * sigma_imm)
sigma_imm ~ dunif(0, 5)

#############################
# Observation process
#############################
# Poisson state-space model
for (t in 1:nyears){
        # juveniles
```

```
        # year-specific average probability among sexes
        p_ave_juv[t] <- (p[t]+p_m[1,t])/2 #part of eq. S54
        counts_juv[t] ~ dbin(p_ave_juv[t], N_juv[t]) #eq. S54
        # non-reproductive individuals (older than juveniles)
        p_ave_nr[t] <- (p[t]+p_m[2,t]+p_m[3,t])/3 #part of eq. S55
        counts_NR[t] ~ dbin(p_ave_nr[t], N_nr[t]) #eq. S55
        # reproductive females
        counts_RF[t] ~ dbin(p[t], N_br[t]) #eq. S56
        # reproductive males
        counts_RM[t] ~ dbin(p_m[4,t], N_ad_m[t]) #eq. S57
}



############################################################
# Derived parameters
############################################################
# Population growth rate
for (t in 1:(nyears-1)){
        lambda[t] <- Ntot[t+1] / Ntot[t]
        l.lambda[t] <- log(lambda[t])
}
# Geometric mean
geomean.lambda <- exp((1/(nyears-1))*sum(l.lambda[1:(nyears-1)]))

# Immigration rate
for (t in 1:(nyears-1)){
        #total
        imm_rate[t] <- N_Imm[t+1]/Ntot[t] #eq. S72
        #females
        imm_rate_f[t] <- N_imm_f[t+1]/Ntot_f[t] #eq. S73
        #males
        imm_rate_m[t] <- N_imm_m[t+1]/Ntot_m[t] #eq. S74
}



############################################################
# Capture-recapture data
############################################################
#----------------------------------------
```

```
#---- CMR: multievent FEMALES
#------------------------------------------
for(t in 1:(nyears-1)){
        # STATE PROCESS:  #eq.  S58
        ps[1,t,1]<-phi[t] * (1-psi_JuPB)
        ps[2,t,1]<-0
        ps[3,t,1]<-0
        ps[4,t,1]<-0
        ps[5,t,1]<-0
        ps[6,t,1]<-0
        ps[7,t,1]<-0
        ps[8,t,1]<-0
        ps[1,t,2]<-phi[t] * psi_JuPB
        ps[2,t,2]<-phi[t] * (1-psi_PBBy)
        ps[3,t,2]<-0
        ps[4,t,2]<-0
        ps[5,t,2]<-0
        ps[6,t,2]<-0
        ps[7,t,2]<-0
        ps[8,t,2]<-0
        ps[1,t,3]<-0
        ps[2,t,3]<-phi[t] * psi_PBBy
        ps[3,t,3]<-0
        ps[4,t,3]<-0
        ps[5,t,3]<-0
        ps[6,t,3]<-0
        ps[7,t,3]<-phi[t] * gamma[t]
        ps[8,t,3]<-0
        ps[1,t,4]<-0
        ps[2,t,4]<-0
        ps[3,t,4]<-phi[t] * (phi_By[t])
        ps[4,t,4]<-0
        ps[5,t,4]<-0
        ps[6,t,4]<-0
        ps[7,t,4]<-0
        ps[8,t,4]<-0
        ps[1,t,5]<-0
        ps[2,t,5]<-0
        ps[3,t,5]<-0
        ps[4,t,5]<-phi[t] * (phi_Bc[t])
```

```
ps[5,t,5]<-0
ps[6,t,5]<-0
ps[7,t,5]<-0
ps[8,t,5]<-0
ps[1,t,6]<-0
ps[2,t,6]<-0
ps[3,t,6]<-0
ps[4,t,6]<-0
ps[5,t,6]<-phi[t] * (phi_Bc[t])
ps[6,t,6]<-0
ps[7,t,6]<-0
ps[8,t,6]<-0
ps[1,t,7]<-0
ps[2,t,7]<-0
ps[3,t,7]<-phi[t] * (1-phi_By[t])
ps[4,t,7]<-phi[t] * (1-phi_Bc[t])
ps[5,t,7]<-phi[t] * (1-phi_Bc[t])
ps[6,t,7]<-phi[t]
ps[7,t,7]<-phi[t] * (1-gamma[t])
ps[8,t,7]<-0
ps[1,t,8]<-(1-phi[t])
ps[2,t,8]<-(1-phi[t])
ps[3,t,8]<-(1-phi[t])
ps[4,t,8]<-(1-phi[t])
ps[5,t,8]<-(1-phi[t])
ps[6,t,8]<-(1-phi[t])
ps[7,t,8]<-(1-phi[t])
ps[8,t,8]<-1

# OBSERVATION PROCESS:  #eq.  S59
po[1,t,1]<-(1-p[t])
po[2,t,1]<-(1-p[t])
po[3,t,1]<-(1-p[t])
po[4,t,1]<-(1-p[t])
po[5,t,1]<-(1-p[t])
po[6,t,1]<-(1-p[t])
po[7,t,1]<-(1-p[t])
po[8,t,1]<-1
po[1,t,2]<-p[t]
po[2,t,2]<-0
```

```
po[3,t,2]<-0
po[4,t,2]<-0
po[5,t,2]<-0
po[6,t,2]<-0
po[7,t,2]<-0
po[8,t,2]<-0
po[1,t,3]<-0
po[2,t,3]<-p[t] * delta
po[3,t,3]<-0
po[4,t,3]<-0
po[5,t,3]<-0
po[6,t,3]<-0
po[7,t,3]<-0
po[8,t,3]<-0
po[1,t,4]<-0
po[2,t,4]<-0
po[3,t,4]<-p[t]
po[4,t,4]<-0
po[5,t,4]<-0
po[6,t,4]<-0
po[7,t,4]<-0
po[8,t,4]<-0
po[1,t,5]<-0
po[2,t,5]<-0
po[3,t,5]<-0
po[4,t,5]<-p[t]
po[5,t,5]<-p[t]
po[6,t,5]<-p[t]
po[7,t,5]<-0
po[8,t,5]<-0
po[1,t,6]<-0
po[2,t,6]<-0
po[3,t,6]<-0
po[4,t,6]<-0
po[5,t,6]<-0
po[6,t,6]<-0
po[7,t,6]<-p[t] * delta
po[8,t,6]<-0
po[1,t,7]<-0
po[2,t,7]<-p[t] * (1-delta)
```

```
        po[3,t,7]<-0
        po[4,t,7]<-0
        po[5,t,7]<-0
        po[6,t,7]<-0
        po[7,t,7]<-p[t] * (1-delta)
        po[8,t,7]<-0
} #t

# Likelihood
for(i in 1:nind){
        z[i,first[i]] <- zFirst[i]
        for (t in (first[i]+1):nyears){
                z[i,t] ~ dcat(ps[z[i,t-1],t-1,1:8])
                y[i,t] ~ dcat(po[z[i,t],t-1,1:7])
        }
}

# PRIORS
for(t in 1:(nyears-1)){
        # survival
        logit(phi[t]) <- mu_phi + eps[t,1] #eq.  S62
        logit(phi_By[t]) <- mu_phi_By + eps[t,2] #eq.  S63
        logit(phi_Bc[t]) <- mu_phi_Bc + eps[t,3] #eq.  S64
        # breeding
        logit(gamma[t]) <- mu_gamma + eps[t,4] #eq.  S65
}
mu_phi <- log(mean_phi / (1-mean_phi))
mean_phi ~ dunif(0, 1)
mu_phi_By <- log(mean_phi_By / (1-mean_phi_By))
mean_phi_By ~ dunif(0, 1)
mu_phi_Bc <- log(mean_phi_Bc / (1-mean_phi_Bc))
mean_phi_Bc ~ dunif(0, 1)
mu_gamma <- log(mean_gamma / (1-mean_gamma))
mean_gamma ~ dunif(0, 1)

# Priors for random effects of demographic rates
for (i in 1:4) {zero[i] <- 0}
        for (t in 1:(nyears-1)){
                eps[t,1:4] ~ dmnorm(zero[], OmegaVCV[,])
}
```

```
# Prior for precision matrix (see variance-covariance matrix in eq.  S66)
OmegaVCV[1:4,1:4] ~ dwish(RR[,],5)
Sigma[1:4,1:4] <- inverse(OmegaVCV[,])

# transition
psi_JuPB ~ dunif(0, 1)
psi_PBBy ~ dunif(0, 1)

# detectability
for(t in 1:nyears){
        logit(p[t]) <- mu_p + beta_p * effort[t] + eps_p[t] #eq.  S70
        eps_p[t] ~ dnorm(0, tau_p)
}
mu_p <- log(mean_p / (1-mean_p))
mean_p ~ dunif(0,1)
beta_p ~ dunif(-5,5)
delta ~ dunif(0, 1)
tau_p <- 1 / (sigma_p * sigma_p)
sigma_p ~ dunif(0, 5)


#-----------------------------------------
#---- CMR: multievent MALES
#-----------------------------------------
for(t in 1:(nyears-1)){
        # STATE PROCESS:  #eq.  S60
        ps_m[1,t,1]<-phi_JS_m[t] * (1-psi_m)
        ps_m[2,t,1]<-0
        ps_m[3,t,1]<-0
        ps_m[4,t,1]<-0
        ps_m[5,t,1]<-0
        ps_m[1,t,2]<-phi_JS_m[t] * psi_m
        ps_m[2,t,2]<-phi_JS_m[t] * (1-psi_m)
        ps_m[3,t,2]<-0
        ps_m[4,t,2]<-0
        ps_m[5,t,2]<-0
        ps_m[1,t,3]<-0
        ps_m[2,t,3]<-phi_JS_m[t] * psi_m
        ps_m[3,t,3]<-phi_Ad_m[t] * (1-psi_m)
```

```
ps_m[4,t,3]<-0
ps_m[5,t,3]<-0
ps_m[1,t,4]<-0
ps_m[2,t,4]<-0
ps_m[3,t,4]<-phi_Ad_m[t] * psi_m
ps_m[4,t,4]<-phi_Ad_m[t]
ps_m[5,t,4]<-0
ps_m[1,t,5]<-(1-phi_JS_m[t])
ps_m[2,t,5]<-(1-phi_JS_m[t])
ps_m[3,t,5]<-(1-phi_Ad_m[t])
ps_m[4,t,5]<-(1-phi_Ad_m[t])
ps_m[5,t,5]<-1
# OBSERVATION PROCESS #eq.  S61
po_m[1,t,1]<-(1-p_m[1,t])
po_m[2,t,1]<-(1-p_m[2,t])
po_m[3,t,1]<-(1-p_m[3,t])
po_m[4,t,1]<-(1-p_m[4,t])
po_m[5,t,1]<-1
po_m[1,t,2]<-p_m[1,t]
po_m[2,t,2]<-0
po_m[3,t,2]<-0
po_m[4,t,2]<-0
po_m[5,t,2]<-0
po_m[1,t,3]<-0
po_m[2,t,3]<-p_m[2,t]
po_m[3,t,3]<-0
po_m[4,t,3]<-0
po_m[5,t,3]<-0
po_m[1,t,4]<-0
po_m[2,t,4]<-0
po_m[3,t,4]<-p_m[3,t] * delta_NT_m
po_m[4,t,4]<-0
po_m[5,t,4]<-0
po_m[1,t,5]<-0
po_m[2,t,5]<-0
po_m[3,t,5]<-0
po_m[4,t,5]<-p_m[4,t] * delta_T_m
po_m[5,t,5]<-0
po_m[1,t,6]<-0
po_m[2,t,6]<-0
```

```
        po_m[3,t,6]<-p_m[3,t] * (1-delta_NT_m)
        po_m[4,t,6]<-p_m[4,t] * (1-delta_T_m)
        po_m[5,t,6]<-0
} #t

# Likelihood
for(i in 1:nind_m){
        z_m[i,first_m[i]] <- zFirst_m[i]
        for (t in (first_m[i]+1):nyears){
                z_m[i,t] ~ dcat(ps_m[z_m[i,t-1],t-1,1:5])
                y_m[i,t] ~ dcat(po_m[z_m[i,t],t-1,1:6])
        }
}

# PRIORS
# survival
for(t in 1:(nyears-1)){
        logit(phi_JS_m[t]) <- mu_phi_JS_m + eps_m[t,1] #eq.  S67
        logit(phi_Ad_m[t]) <- mu_phi_Ad_m + eps_m[t,2] #eq.  S68
}
mu_phi_JS_m <- log(mean_phi_JS_m / (1-mean_phi_JS_m))
mean_phi_JS_m ~ dunif(0, 1)
mu_phi_Ad_m <- log(mean_phi_Ad_m / (1-mean_phi_Ad_m))
mean_phi_Ad_m ~ dunif(0, 1)

# Priors for random effects of demographic rates
for (i in 1:2) {zero_m[i] <- 0}
        for (t in 1:(nyears-1)){
                eps_m[t,1:2] ~ dmnorm(zero_m[], OmegaVCV_m[,])
}

# Prior for precision matrix (see variance-covariance matrix in eq.  S69)
OmegaVCV_m[1:2,1:2] ~ dwish(RR_m[,],3)
Sigma_m[1:2,1:2] <- inverse(OmegaVCV_m[,])

# transition
psi_m ~ dunif(0,1)

# detectability
for(t in 1:nyears){
```

```
        for(c in 1:4){
                logit(p_m[c,t]) <- mu_p_m + stageEff[c] +
                                   beta_p_m * effort[t] + eps_p_m[t] #eq.  S71
        }
        eps_p_m[t] ~ dnorm(0, tau_p_m)
}
mu_p_m <- log(mean_p_m / (1-mean_p_m))
mean_p_m ~ dunif(0,1)
beta_p_m ~ dunif(-5,5)
tau_p_m <- 1 / (sigma_p_m * sigma_p_m)
sigma_p_m ~ dunif(0, 5)
# corner constraint
stageEff[1] <- 0
for(c in 2:4){
        stageEff[c] ~ dunif(-5,5)
}

delta_NT_m ~ dunif(0,1)
delta_T_m ~ dunif(0,1)

} # end of model
```

## S.5 R code to run the integrated population model in JAGS

```
# set working directory
setwd("~/your/path/")

# Load R libraries
library(jagsUI)
library(coda)

#-------------------------------------------
#----------- CMR: multievent FEMALES
#-------------------------------------------
# Read in the data:
CH_f <- read.table(file='CH_Female_Multi_2004_6events_v3.txt',header=T,sep="␣")
head(CH_f)
dim(CH_f)
nind <- dim(CH_f)[1]
nyears <- dim(CH_f)[2]

# Derive first capture for each individual:
first <- NULL
for (i in 1:nind){
temp <- 1:nyears
first <- c(first,min(temp[CH_f[i,]>=1]))}

# OBSERVATIONS (CH_m + 1)
# 1 = not seen
# 2 = seen as juvenile
# 3 = seen as pre-breeder
# 4 = seen as breeder with a young-of-the-year
# 5 = seen as breeder with a 1-year-old or older calf
# 6 = seen as non-breeder
# 7 = seen alone

# STATES
# JUV: juvenile
# PB: pre-breeder
# By: breeder with a young-of-the-year
# Bc1: breeder with a 1-year-old calf
# Bc2: breeder with a 2-year-old calf
```

```
# Bc3: breeder with a 3-year-old calf
# NB: non-breeder
# D: dead

# initial values for latent z states and state at first encounter
z_inits <- as.matrix(read.table(file='CH_Female_Multi_2004_z_init_Nimble.txt',header=T,sep="␣"))

# state at first encounter
zFirst <- NULL
for(i in 1:nind){
        zFirst[i] <- z_inits[i,first[i]]
}

# initial values for JAGS
z_inits_2 <- z_inits

for(i in 1:nind){
for(t in 1:nyears){
        if(first[i]==1){z_inits_2[i,first[i]] <- NA}
        else{z_inits_2[i,1:first[i]] <- NA}
}
}



#-----------------------------------------
#---------- CMR: multievent MALES
#-----------------------------------------
# Read in the data:
CH_m <- read.table(file='CH_headed_Male_Multi_2004.txt',header=T,sep="␣")
head(CH_m)
dim(CH_m)
nind_m <- dim(CH_m)[1]

# Derive first capture for each individual:
first_m <- NULL
for (i in 1:nind_m){
temp_m <- 1:nyears
first_m <- c(first_m,min(temp_m[CH_m[i,]>=1]))}

# OBSERVATIONS (CH_m + 1)
```

```
#1 = not seen
#2 = seen as juvenile
#3 = seen as subadult
#4 = seen as not toothed adult
#5 = seen as toothed adult
#6 = seen as adult (teeth not ascertained)

# STATES
# JUV: juvenile
# SUB: subadult
# AD_nt: not toothed adult male
# AD_t: toothed adult male
# D: dead

# initial values for latent z states and state at first encounter
z_inits_m <- as.matrix(read.table(file='CH_headed_Male_Multi_2004_z_init.txt',header=T,sep="␣"))

# state at first encounter
zFirst_m <- NULL
for(i in 1:nind_m){
        zFirst_m[i] <- z_inits_m[i,first_m[i]]
}

# initial values for JAGS
z_inits_m_2 <- z_inits_m

for(i in 1:nind_m){
for(t in 1:nyears){
        if(first_m[i]==1){z_inits_m_2[i,first_m[i]] <- NA}
        else{z_inits_m_2[i,1:first_m[i]] <- NA}
}
}

#---------- Sampling effort (standardized)
effort <- c(1.2155930,1.7251866,1.5553221,0.4512026,-1.7570362,-0.6529168,
            -1.1625103,-0.8227813,-0.6529168,0.2813381,-0.1433232,0.1964059,
            -0.9077136,-0.4830522,0.7909317,0.3662704)

#---------- generate discrete unifor probabilities
disc.unif <- function(A, B){
```

```
        pprob <- c(rep(0, A-1), rep(1/(B-A+1), (B-A+1)))
        return(pprob)
        }

#----------- JAGS data
# Count data
load("count_data.RData")

bugs.data <- list(
                  # CMR Multievent FEMALES
                  y=as.matrix(CH_f+1),first=first,nyears=nyears,nind=nind,
                  zFirst=zFirst,effort=effort,
                  RR=diag(c(1,1,1,1)),
                  # CMR Multievent MALES
                  y_m=as.matrix(CH_m+1),first_m=first_m,nind_m=nind_m,
                  zFirst_m=zFirst_m,
                  RR_m=diag(c(1,1)),
                  # State space
                  counts_juv=counts_juv,counts_NR=counts_NR,counts_RF=counts_RF,
                  counts_RM=counts_RM,
                  # FEMALES
                  #total number of juveniles from individuals weaned between
                  #2nd and 3rd year of life
                  p_n_juv_tot_part1a_inits=disc.unif(2,30),
                  #number of juveniles from 3yr weaned
                  p_n_juv_f_part1b_inits=disc.unif(2,25),
                  #number of dead calves between 2nd and 3rd year of life (mother alive)
                  p_n_dead23_inits=disc.unif(2,20),
                  #number of juveniles from juveniels of previous year
                  p_n_juv_f_part2_inits=disc.unif(2,30),
                  #number of pre-breeders
                  p_n_pb_f_part1_inits=disc.unif(2,25),
                  p_n_pb_f_part2_inits=disc.unif(2,35),
                  #number of breeders with young of the year
                  p_n_by_f_part1_inits=disc.unif(2,15),
                  p_n_by_f_part2_inits=disc.unif(2,20),
                  #number of breeders with a 1yr calf
                  p_n_bc1_f_inits=disc.unif(2,15),
                  #number of breeders with a 2yr calf
                  p_n_bc2_f_inits=disc.unif(2,30),
```

```
                 #number of breeders with a 3yr calf
                 p_n_bc3_f_inits=disc.unif(2,15),
                 #number of non-breeders
                 p_n_nb_f_part1b_inits=disc.unif(2,32),
                 p_n_nb_f_part1d_inits=disc.unif(1,5),
                 p_n_nb_f_part1e_inits=disc.unif(1,5),
                 p_n_nb_f_part2_inits=disc.unif(2,22),
                 # MALES
                 #number of juveniles from 3yr weaned
                 p_n_juv_m_part1b_inits=disc.unif(2,25),
                 #number of juveniles from juveniels of previous year
                 p_n_juv_m_part2_inits=disc.unif(2,25),
                 # number of subadults
                 p_n_sub_m_part1_inits=disc.unif(2,20),
                 p_n_sub_m_part2_inits=disc.unif(2,30),
                 # number of adults not toothed
                 p_n_adnt_m_part1_inits=disc.unif(2,20),
                 p_n_adnt_m_part2_inits=disc.unif(6,30),
                 # number of toothed adults
                 p_n_ad_m_part1_inits=disc.unif(2,15),
                 p_n_ad_m_part2_inits=disc.unif(2,25),
                 # TOTAL number of immigrants
                 p_n_imm_inits=disc.unif(10,50)
                )

#----------- Parameters to monitor
parameters <- c(
               # CMR Multievent FEMALES
               "phi","phi_By","phi_Bc","mean_phi","mean_phi_By","mean_phi_Bc","Sigma",
               "psi_JuPB","psi_PBBy","mean_gamma","gamma","p",
               "mean_p","beta_p","delta","sigma_p",
               # CMR Multievent MALES
               "phi_JS_m","phi_Ad_m","mean_phi_JS_m","mean_phi_Ad_m","Sigma_m",
               "psi_m",
               "p_m","mean_p_m","beta_p_m","sigma_p_m","stageEff","delta_NT_m","delta_T_m",
               # Immigration
               "mean.omega_imm","sigma_imm","imm_rate","imm_rate_f","imm_rate_m",
               # Growth rate
               "lambda", "geomean.lambda",
               # Population sizes (state process)
```

```
                    "fid","N_juv_tot_part1a","N_juv_f_part1a","N_w3","N_w3_f","N_w3_m",
                    "Ndead23","Ndead_f","N_juv_f_part1b","N_juv_f_part2",
                    "pi1","pi2","pi3","Npi2","Npi3",
                    "N_juv_f_NoImm","N_juv_f","N_pb_f_part1","N_pb_f_part2","N_pb_f",
                    "N_by_f_part1","N_by_f_part2","N_by_f",
                    "N_bc1_f","N_bc2_f","N_bc3_f",
                    "N_nb_f_part1a","N_nb_f_part1b","N_nb_f_part1c","N_nb_f_part1d",
                    "N_nb_f_part1e","N_nb_f_part2","N_nb_f","N_imm_f",
                    #
                    "N_juv_m_part1a","N_juv_m_part1b","N_juv_m_part2",
                    "N_juv_m_NoImm","N_juv_m","N_sub_m_part1","N_sub_m_part2","N_sub_m",
                    "N_adnt_m_part1","N_adnt_m_part2","N_adnt_m",
                    "N_ad_m_part1","N_ad_m_part2","N_ad_m","N_imm_m",
                    "Ntot_f","Ntot_m",
                    "Ntot","N_juv","N_Imm","N_nr","N_br",
                    # Population sizes (observation process)
                    "p_ave_juv","p_ave_nr"
                   )


#----------- Initial values
######### initial population sizes
#total number of juveniles from individuals weaned between 2nd and 3rd year of life
N_juv_tot_part1a <- rep(NA, nyears)
N_juv_tot_part1a[1] <- 18
#----------------------------------------
#---- FEMALES
#----------------------------------------
#number of juveniles from 3yr weaned
N_juv_f_part1b <- rep(NA, nyears)
N_juv_f_part1b[1] <- 6
#number of dead calves between 2nd and 3rd year of life (mother alive)
Ndead23 <- rep(NA, nyears)
Ndead23[1] <- 5
#number of juveniles from juveniels of previous year
N_juv_f_part2 <- rep(NA, nyears)
N_juv_f_part2[1] <- 25
#number of pre-breeders
N_pb_f_part1 <- rep(NA, nyears)
N_pb_f_part2 <- rep(NA, nyears)
```

```
N_pb_f_part1[1] <- 20
N_pb_f_part2[1] <- 30
#number of breeders with young of the year
N_by_f_part1 <- rep(NA, nyears)
N_by_f_part2 <- rep(NA, nyears)
N_by_f_part1[1] <- 10
N_by_f_part2[1] <- 14
#number of breeders with a 1yr calf
N_bc1_f <- rep(NA, nyears)
N_bc1_f[1] <- 14
#number of breeders with a 2yr calf
N_bc2_f <- rep(NA, nyears)
N_bc2_f[1] <- 30
#number of breeders with a 3yr calf
N_bc3_f <- rep(NA, nyears)
N_bc3_f[1] <- 8
#number of non-breeders
N_nb_f_part1b <- rep(NA, nyears)
N_nb_f_part1d <- rep(NA, nyears)
N_nb_f_part1e <- rep(NA, nyears)
N_nb_f_part2 <- rep(NA, nyears)
N_nb_f_part1d[1] <- 1
N_nb_f_part1e[1] <- 1
N_nb_f_part1b[1] <- 30
N_nb_f_part2[1] <- 20
#----------------------------------------
#---- MALES
#----------------------------------------
#number of juveniles
N_juv_m_part1b <- rep(NA, nyears)
N_juv_m_part2 <- rep(NA, nyears)
N_juv_m_part1b[1] <- 6
N_juv_m_part2[1] <- 25
# number of subadults
N_sub_m_part1 <- rep(NA, nyears)
N_sub_m_part2 <- rep(NA, nyears)
N_sub_m_part1[1] <- 15
N_sub_m_part2[1] <- 25
# number of adults not toothed
N_adnt_m_part1 <- rep(NA, nyears)
```

```
N_adnt_m_part2 <- rep(NA, nyears)
N_adnt_m_part1[1] <- 15
N_adnt_m_part2[1] <- 25
# number of toothed adults
N_ad_m_part1 <- rep(NA, nyears)
N_ad_m_part2 <- rep(NA, nyears)
N_ad_m_part1[1] <- 10
N_ad_m_part2[1] <- 20
# TOTAL number of immigrants
N_imm <- rep(NA, nyears)
N_imm[1] <- 40

inits <- function(){list(
                         # CMR Multievent FEMALES
                         OmegaVCV = diag(4),mean_phi=runif(1,0.97,0.98),
                         mean_phi_By=runif(1,0.88,0.90),mean_phi_Bc=runif(1,0.28,0.29),
                         psi_JuPB=runif(1,0.41,0.42),psi_PBBy=runif(1,0.22,0.24),
                         mean_gamma=runif(1,0.30,0.40),
                         mean_p=runif(1,0.19,0.20),beta_p=runif(1,0.59,0.60),
                         sigma_p=runif(1,0.9,1),delta=runif(1,0.64,0.65),
                         z = z_inits_2,
                         # CMR Multievent MALES
                         OmegaVCV_m=diag(2),mean_phi_JS_m=runif(1,0.98,0.99),
                         mean_phi_Ad_m=runif(1,0.94,0.95),psi_m=runif(1,0.24,0.25),
                         mean_p_m=runif(1,0.18,0.19),beta_p_m=runif(1,0.66,0.68),
                         sigma_p_m=runif(1,0.92,0.93),
                         stageEff=c(NA,runif(1,1.35,1.37),
                                    runif(1,1.05,1.06),runif(1,0.55,0.56)),
                         delta_NT_m=runif(1,0.62,0.63),delta_T_m=runif(1,0.96,0.97),
                         z_m = z_inits_m_2,
                         # Initial population sizes
                         N_juv_tot_part1a=N_juv_tot_part1a,
                         N_juv_f_part1b=N_juv_f_part1b,
                         Ndead23=Ndead23,
                         N_juv_f_part2=N_juv_f_part2,
                         N_pb_f_part1=N_pb_f_part1,
                         N_pb_f_part2=N_pb_f_part2,
                         N_by_f_part1=N_by_f_part1,
                         N_by_f_part2=N_by_f_part2,
                         N_bc1_f=N_bc1_f,
```

```
                        N_bc2_f=N_bc2_f,
                        N_bc3_f=N_bc3_f,
                        N_nb_f_part1b=N_nb_f_part1b,
                        N_nb_f_part1d=N_nb_f_part1d,
                        N_nb_f_part1e=N_nb_f_part1e,
                        N_nb_f_part2=N_nb_f_part2,
                        N_juv_m_part1b=N_juv_m_part1b,
                        N_juv_m_part2=N_juv_m_part2,
                        N_sub_m_part1=N_sub_m_part1,
                        N_sub_m_part2=N_sub_m_part2,
                        N_adnt_m_part1=N_adnt_m_part1,
                        N_adnt_m_part2=N_adnt_m_part2,
                        N_ad_m_part1=N_ad_m_part1,
                        N_ad_m_part2=N_ad_m_part2,
                        N_imm=N_imm
                        )}


#----------- MCMC settings
n.adapt <-    100000
n.burnin <-   3000000
n.iter <-     n.burnin+300000
thin <-       15
chains <-     4



#----------- Run the model
out <- jags(data = bugs.data,
                parameters.to.save = parameters,
                model = "IPM_10.txt", #model script in Section S.4
                inits = inits,
                n.adapt = n.adapt,
                n.burnin = n.burnin,
                n.iter = n.iter,
                n.thin = thin,
                n.chains = chains,
                parallel=T)
# end of script
```

# REFERENCES

Abadi, F., O. Gimenez, R. Arlettaz, and M. Schaub, 2010. An assessment of integrated population models: bias, accuracy, and violation of the assumption of independence. *Ecology* **91**:7–14.

Alvarez, I., J. Niemi, and M. Simpson, 2014. Bayesian inference for a covariance matrix. *arXiv preprint arXiv:1408.4050* .

Brooks, S. P., E. A. Catchpole, B. J. Morgan, and S. Barry, 2000. On the bayesian analysis of ring-recovery data. *Biometrics* **56**:951–956.

Choquet, R., A. Reboulet, J. Lebreton, O. Gimenez, and R. Pradel, 2005. U-CARE 2.2, user's manual. CEFE.

Choquet, R., L. Rouan, and R. Pradel, 2009. Program e-surge: A software application for fitting multievent models. In *Environmental and Ecological Statistics. Edited by Thomson, D., Cooch, E. and Conroy, M.*, volume 3, pages 845865–845865. Springer.

Cormack, R. M., 1964. Estimates of survival from the sighting of marked animals. *Biometrika* **51**:429–438.

Couet, P., F. Gally, C. Canonne, and A. Besnard, 2019. Joint estimation of survival and breeding probability in female dolphins and calves with uncertainty in state assignment. *Ecology and evolution* **9**:13043–13055.

Gelman, A. and J. Hill, 2006. Data Analysis Using Regression and Multilevel/Hierarchical Models. Cambridge University Press.

Gelman, A., X.-L. Meng, and H. Stern, 1996. Posterior predictive assessment of model fitness via realized discrepancies. *Statistica sinica* pages 733–760.

Gimenez, O., J.-D. Lebreton, R. Choquet, and R. Pradel, 2017. R2ucare: Goodness-of-Fit Tests for Capture-Recapture Models. R package version 1.0.0.

Grosbois, V. and G. Tavecchia, 2003. Modeling dispersal with capture-recapture data: disentangling decisions of leaving and settlement. *Ecology* **84**:1225–1236.

Jolly, G. M., 1965. Explicit estimates from capture-recapture data with both death and immigration-stochastic model. *Biometrika* **52**:225–247.

Kéry, M. and J. A. Royle, 2015. Applied Hierarchical Modeling in Ecology: Analysis of distribution, abundance and species richness in R and BUGS: Volume 1: Prelude and Static Models. Academic Press.

Koons, D. N., T. W. Arnold, and M. Schaub, 2017. Understanding the demographic drivers of realized population growth rates. *Ecological Applications* **27**:2102–2115.

Koons, D. N., D. T. Iles, M. Schaub, and H. Caswell, 2016. A life-history perspective on the demographic drivers of structured population dynamics in changing environments. *Ecology letters* **19**:1023–1031.

Pradel, R., 2005. Multievent: an extension of multistate capture-recapture models to uncertain states. *Biometrics* **61**:442–447.

Schaub, M., H. Jakober, and W. Stauber, 2013. Strong contribution of immigration to local population regulation: evidence from a migratory passerine. *Ecology* **94**:1828–1838.

Seber, G. A., 1965. A note on the multiple-recapture census. *Biometrika* **52**:249–259.

Weegman, M. D., T. W. Arnold, R. G. Clark, and M. Schaub, 2020. Partial and complete dependency among data sets has minimal consequence on estimates from integrated population models. *Ecological Applications* page e2258.
