# Supplementary material for: Immigration as the main driver of population dynamics in a cryptic cetacean
Source: Ecol Evol. 2023 Feb 11;13(2):e9806. doi: 10.1002/ece3.9806 (PMC9919498; doi:10.1002/ece3.9806)
Supplement: Supplementary file 2 — Appendix S2. [file ECE3-13-e9806-s001.pdf]

Simone Tenan, Aurelie Moulins, Paola Tepsich, Alessandro Bocconcelli, Alessandro Verga, Marco Ballardini, Barbara Nani, Daniela Papi, Gabriella Motta, Ana Sanz Aguilar, and Massimiliano Rosso

Immigration as the main driver of population dynamics in a cryptic cetacean

Appendix S2: Details on parameter estimates.

Table S1: Posterior estimates (mean, standard deviation, and percentiles) for the demographic parameters of the Cuvier's beaked whale population.

Parameter *	mean	SD	2.5%	50%	97.5%
$\phi_{0,F}$	0.980	0.014	0.946	0.982	0.998
$\phi_{0,By,F}$	0.835	0.098	0.602	0.851	0.973
$\phi_{0,Bc,F}$	0.256	0.118	0.070	0.243	0.524
$\psi_{JuvPB,F}$	0.245	0.078	0.121	0.235	0.425
$\psi_{PbBy,F}$	0.218	0.052	0.127	0.214	0.327
γ_0	0.437	0.105	0.238	0.435	0.648
σ_{ϕ_F}	1.145	0.657	0.389	0.972	2.829
$\sigma_{\phi_{By}}$	1.927	0.965	0.563	1.759	4.323
$\sigma_{\phi_{Bc}}$	1.570	0.829	0.479	1.403	3.659
σ_γ	0.885	0.449	0.364	0.771	2.072
$p_{0,F}$	0.183	0.045	0.109	0.179	0.285
β_{p_F}	0.702	0.285	0.139	0.699	1.280
δ_F	0.643	0.063	0.514	0.645	0.763
σ_{p_F}	0.973	0.285	0.537	0.934	1.646
$\phi_{0,JuvSub,M}$	0.989	0.010	0.963	0.992	1.000
$\phi_{0,Ad,M}$	0.949	0.016	0.915	0.950	0.978
ψ_M	0.250	0.026	0.201	0.249	0.304
$\sigma_{\phi_{JuvSub,M}}$	0.799	0.396	0.347	0.698	1.837
$\sigma_{\phi_{Ad,M}}$	0.754	0.332	0.347	0.676	1.609
$p_{0,M}$	0.292	0.083	0.150	0.284	0.475
β_{p_M}	0.647	0.274	0.107	0.645	1.199
σ_{p_M}	0.992	0.239	0.630	0.956	1.557
stage_{Sub}	0.687	0.391	-0.075	0.689	1.458
stage_{AdNt}	0.384	0.389	-0.376	0.383	1.153
stage_{Ad}	-0.053	0.365	-0.763	-0.054	0.669
$\delta_{AdNt,M}$	0.625	0.064	0.498	0.627	0.746
$\delta_{Ad,M}$	0.965	0.020	0.918	0.969	0.993

* The parameters are: intercept of female apparent survival probability $\phi_{0,F}$ = inverse-logit(μ_{ϕ_F}); intercept of young-of-the-year survival $\phi_{0,By,F}$ = inverse-logit($\mu_{\phi_{By,F}}$); intercept of apparent calf survival $\phi_{0,Bc,F}$ = inverse-logit($\mu_{\phi_{Bc,F}}$); transition probability from juvenile to pre-breeding female $\psi_{JuvPB,F}$; transition probability from pre-breeding to breeding female (i.e. probability of first reproduction) $\psi_{PbBy,F}$; intercept of breeding probability for non-breeding females γ_0 = inverse-logit(μ_γ); temporal random standard deviation for the above-mentioned survival and breeding probability (σ_{ϕ_F}, $\sigma_{\phi_{By}}$, $\sigma_{\phi_{Bc}}$, σ_γ); intercept of female encounter probability $p_{0,F}$ = inverse-logit(μ_{p_F}); effect of year-specific sampling effort on female encounter probability β_{p_F}; probability that the state of a female was ascertained during observation δ_F; encounter probability

temporal random standard deviation for females σ_{p_F}; intercept of apparent survival probability for juvenile and subadult males $\phi_{0,JuvSub,M,t}$ = inverse-logit($\mu_{\phi_{JuvSub,M}}$); intercept of apparent survival probability for adult males (both not toothed and toothed) $\phi_{0,Ad,M}$ = inverse-logit($\mu_{\phi_{Ad,M}}$); male transition probability ψ_M; temporal random standard deviation for the male apparent survival probabilities ($\sigma_{\phi_{JuvSub,M}}$, $\sigma_{\phi_{Ad,M}}$); intercept of male encounter probability $p_{0,M}$ = inverse-logit(μ_{p_M}); effect of year-specific sampling effort on male encounter probability β_{p_M}; encounter probability temporal random standard deviation for males σ_{p_M}; effect of stage on male probabilities (with stage$_{Juv}$ as reference level); probabilities that the state of was ascertained during observation, for not toothed and toothed adult males ($\delta_{AdNt,M}$ and $\delta_{Ad,M}$, respectively). Intercepts of survival, breeding, and encounter probabilities, transition probabilities, and probabilities that the state of was ascertained during observation are given on the probability scale. Effect of sampling effort and stage on encounter probability, as well as all σs, are on logit scale.

Table S2: Posterior means of temporal correlation coefficients between female demographic rates (above diagonal; ρ_{1-6} in Appendix S1 Eqs. S66) and probabilities that correlation coefficients are positive $[P(\rho_{1-6} > 0)]$ (below diagonal). Note that the probability of a negative correlation is $1 - P(\rho_{1-6} > 0)$.

	σ_{ϕ_F}	$\sigma_{\phi_{By}}$	$\sigma_{\phi_{Bc}}$	σ_γ
σ_{ϕ_F}		-0.29	-0.27	-0.06
$\sigma_{\phi_{By}}$	0.31		0.70	0.08
$\sigma_{\phi_{Bc}}$	0.32	0.94		-0.12
σ_γ	0.45	0.56	0.41	

Table S3: Posterior mean of the temporal correlation coefficient between male demographic rates (above diagonal; ρ_7 in Appendix S1 Eqs. S69) and the probability that the correlation coefficient is positive $[P(\rho_7 > 0)]$ (below diagonal). Note that the probability of a negative correlation is $1 - P(\rho_7 > 0)$.

	$\sigma_{\phi_{JuvSub,M}}$	$\sigma_{\phi_{Ad,M}}$
$\sigma_{\phi_{JuvSub,M}}$		-0.02
$\sigma_{\phi_{Ad,M}}$	0.48	
